# GHGs and air pollutants embodied in China’s international trade: Temporal and spatial index decomposition analysis

**DOI:** 10.1371/journal.pone.0176089

**Published:** 2017-04-25

**Authors:** Zhengyan Liu, Xianqiang Mao, Peng Song

**Affiliations:** 1 School of Environment, Beijing Normal University, Beijing, P. R. China; 2 School of Public Affairs, Chongqing University, Chongqing, P. R. China; Universita degli Studi della Tuscia, ITALY

## Abstract

Temporal index decomposition analysis and spatial index decomposition analysis were applied to understand the driving forces of the emissions embodied in China’s exports and net exports during 2002–2011, respectively. The accumulated emissions embodied in exports accounted for approximately 30% of the total emissions in China; although the contribution of the sectoral total emissions intensity (technique effect) declined, the scale effect was largely responsible for the mounting emissions associated with export, and the composition effect played a largely insignificant role. Calculations of the emissions embodied in net exports suggest that China is generally in an environmentally inferior position compared with its major trade partners. The differences in the economy-wide emission intensities between China and its major trade partners were the biggest contribution to this reality, and the trade balance effect played a less important role. However, a lower degree of specialization in pollution intensive products in exports than in imports helped to reduce slightly the emissions embodied in net exports. The temporal index decomposition analysis results suggest that China should take effective measures to optimize export and supply-side structure and reduce the total emissions intensity. According to spatial index decomposition analysis, it is suggested that a more aggressive import policy was useful for curbing domestic and global emissions, and the transfer of advanced production technologies and emission control technologies from developed to developing countries should be a compulsory global environmental policy option to mitigate the possible leakage of pollution emissions caused by international trade.

## 1. Introduction

China has achieved rapid economic growth since its opening-up, especially after it became a member of the World Trade Organization (WTO) in 2001. During the 10-year period from 2002 to 2011, which roughly corresponds with the 10^th^ and 11^th^ Five Year Plan (FYP) periods, China’s exports and imports increased by 4.9-fold and 4.7-fold, respectively. International trade accelerated economic development, and China’s gross domestic product (GDP) increased at an annual growth rate of 10.3%. With the rapid growth of its economy and international trade, China has become the largest exporter, the second largest importer and the second largest national economy in the world [1].

As the “world’s factory” during the past decades, China has experienced a tremendous increasing demand for resources and a great discharge of pollutants. In 2010, China accounted for 20% of the global energy demand [2] and surpassed the USA to become the world's largest consumer of energy and the top emitter of greenhouse gases (GHGs), accounting for 23.9% of the world’s total GHGs emissions [3]. Although there was a decline in sulfur dioxide (SO_2_) emissions over the 11^th^ FYP period (2006–2010), the total quantity emitted remained enormous. Nitrous oxide (NO_x_) emissions continued to increase along with a rapid growth in GDP during those years. The discharge volumes of SO_2_ and NO_x_ were 22.2 million tons and 20.4 million tons in 2011, respectively, which were even larger than the total emissions of each of these pollutants in the European Union countries [4].

Given that developing countries, such as China, generally have less efficient production technologies and fewer environmental restrictions, the relocation of labor-intensive manufacturing from developed to developing countries is often considered tantamount to a transfer of fossil fuel and water consumption and environmental impact [5–11]. Usually, industrialized countries are net importers of embodied carbon dioxide (CO_2_) emissions, whereas developing countries, such as China and Russia, are net exporters [12]; in fact, developed countries have experienced an increase in embodied emissions by increasing imports of embodied CO_2_ and traditional air pollutants [13]. Those findings have given rise to considerable discussion regarding the consumption-based accounting of carbon emissions and the potential for international carbon leakage [12, 14–18].

In 2005, it was estimated that nearly 30% of the Chinese CO_2_ emissions were linked to production for export [19–20]. SO_2_ emissions embodied in exports contributed 15.17%–22.08% of the total domestic SO_2_ emissions in 2002–2007 [21]. Xu et al. demonstrated that energy embodied in the exports from China to the USA accounted for approximately 12%-17% of China’s energy consumption, and embodied CO_2_ represented approximately 8%-12% of China’s CO_2_ emissions. SO_2_ and NO_x_ embodied in the exports to the USA accounted for 10%-15% and 8%-12% of China’s total emissions during 2002–2007, respectively [22].

Yan et al. estimated that 10.03%-26.54% of China’s annual CO_2_ emissions were produced during the manufacture of export goods destined for foreign consumers from 1997 to 2007, whereas the CO_2_ emissions embodied in China’s imports (calculated using the USA’s CO_2_ emissions factors) accounted for only 4.40% (1997) and 9.05% (2007) of the annual CO_2_ emissions in China [23]. Ren et al. also demonstrated that the quantity of China's industrial CO_2_ emissions embodied in exports has been considerably larger than those embodied in imports, as determined by utilizing the average emissions factors of the China’s 10 largest trade partners from 2001 to 2011. In addition, these researchers asserted that the carbon emissions embodied in China’s net exports accounted for approximately 30% of the industrial carbon emissions and that China has thus become a pollution haven [24]. The numerical results of the embodied CO_2_ emissions in China’s foreign trade are of great discrepancies within given year by different considerations on methodology specification, accounting principles, and data sources and processing. For instance, the estimates of CO_2_ embodied in China’s exports changed from 478 Mt to over 3000 Mt and those of in China’s imports ranged from 140 Mt to over 1700 Mt in 2007 [25].

Most of these studies provide observations and a description and evaluation of the environmental performance of Chinese international trade. However, what are the major driving forces for the changes of the emissions embodied in trade? How large a contribution have the different driving forces respectively made? Did the huge export trade volume, the high emissions intensity and the net trade balance of China account for the huge domestic emissions? Many studies have been done to identify the driving forces of the emissions embodied in China's trade, either for bilateral [26–29] or multilateral [23, 30–33] trade. Most of these previous studies mainly set emphasis only on temporal driving force analysis or only on the spatial driving force analysis [34–35]. Analysis focused only on temporal or on spatial driving forces cannot fully reflect the internal and external driving forces that actually have been together shaping the changes in the environmental performance indicators of China’s foreign trade. And the rational and comprehensive design of environmentally friendly trade-related policies would not be available if internal and external driving forces are not considered simultaneously [36]. Thus, the present study intends to employ both the temporal and spatial decomposition methods to analyze emissions embodied in trade, namely the emissions embodied in the exports (EEE) and the balance of emissions embodied in trade (BEET), for China.

Meanwhile, most previous studies considered CO_2_ emissions as the sole indicator, which reflects the global concern about the mitigation of climate change. However, other GHG emissions, such as N_2_O and CH_4_, are also of great interest. More realistically, for the domestic general public and environmental policy makers, local or traditional air pollutants, such as SO_x_ and NO_x_, would be of more political relevance. Thus CO_2_, CH_4_, N_2_O, SO_x_ and NO_x_ were chosen as the emissions indicators for this study to fill this gap in the data.

This paper is organized as follows: after the introduction and background, section 2 details the methods and the data used to calculate the trade-embodied emissions and the driving force decomposition approach. Section 3 describes the results of the calculation of the trade-embodied pollutants emissions and the temporal and spatial driving force analysis for China. The policy implication and an uncertainty analysis are then discussed. Finally, the study’s conclusions and several suggestions for environmentally friendly trade-related policies are presented.

## 2. Methods and data

### 2.1 Methods

Single region environmental input-output (SREIO) models were built for China and its major trade partners containing 15 aggregative sectors each based on the World Input–Output Database (WIOD) [37]. Next, the GHGs and air pollutant emissions embodied in China’s exports were calculated based on the SREIO for China, and those embodied in China’s imports were calculated based on the SREIOs of its major trade partners. Additionally, the GHGs and air pollutants emissions embodied in the net exports of China were inferred from the difference of the previous two. The time horizon of the models starts from China’s WTO accession in 2002 and runs for 10 years to 2011. Next, a temporal index decomposition analysis (IDA) and a spatial index decomposition analysis were conducted to derive the driving forces of the emissions embodied in China’s international trade.

#### 2.1.1 Environmental performance indicators of trade and the environmental input-output model

The current study employed the emissions embodied in the exports (EEE) to represent the domestic environmental impact of the exports [21]. The balance of emissions embodied in trade (BEET) is used to denote the difference between the emissions embodied in the imports and in the exports [38], which is similar to the “emission trade balance” (ETB) defined by Arto et al. [39]. The BEET was calculated by subtracting the emissions embodied in the imports (EEI), calculated using the emission factors of the trade partners who produced the imported goods [40] from the EEE.

Environmental input-output (EIO) model is among the most popular methods for quantitatively evaluating the environmental effects of trade [41–45]. EIO models based on both single-region input-output (SRIO) and multi-region input-output (MRIO) models have been utilized by researchers [32, 46–47]. Kanemoto et al. recommended that the SRIO approach should be used due to its consistency with the monetary trade balance to compare trade-adjusted emissions inventories [48]. In the current study, EIO models were employed to calculate the emissions embodied in China’s exports (EEE) and imports (EEI). Because the research was focused on the EEE, EEI and BEET of China, it was not necessary to obtain the emissions transferred among diverse regions via international trade. Instead, single-region EIO models, which address the exports and imports of China, as an open economy, and its major trade partners were adopted.

The total output *x* can be expressed as the sum of the intermediate consumption *Ax* and the final consumption *y*:

x=Ax+y, (1)

The matrix *A* describes the relationship between all sectors of the economy, where *a*_*ij*_ is the element of the matrix *A* that indicates the sector *i* products directly utilized in production by sector *j*. When solved for total output, this equation yields

$$\text{x}={\left(I-A\right)}^{-1}y,$$ (2)

where *I* is the identity matrix, and (*I* − *A*)^−1^ is called “Leontief’s inverse matrix”.

An environmental extension of the basic input–output model can be obtained by introducing the matrix *f*, which includes the pollutant emissions for one unit of monetary output for each sector [9]. Considering the difference between imported and domestically produced goods, *A*^*d*^ was used to represent the direct requirement coefficient matrix of the domestic intermediate input. The product of the environmental matrix *f* and (*I* − *A*^*d*^)^−1^ gives the multiplier matrix *F*, which indicates the domestic total emissions intensity (TEI)–direct plus indirect emissions–for the GHGs and pollutants for each sector:

$$F=f{\left(I-A^{d}\right)}^{-1},$$ (3)

*EEE*, *EEI*, and *BEET* [49] can be calculated as

$$EEE=F_{ex}\times X,$$ (4)

$$EEI=F_{tp}\times M,$$ (5)

BEET=EEE−EEI, (6)

where *F*_*ex*_ and *F*_*tp*_ are the matrices of the sectoral TEIs of the home country—China in this study (the subscript ex denotes export) and its trade partners (the subscript tp denotes the trade partners), respectively, and *X* and *M* are the matrixes of the export and import, respectively, of products and services.

#### 2.1.2 Index decomposition analysis model

After the environmental performance indicators of EEE, EEI and BEET have been calculated using SREIO, the next step is to decompose the indicators into the appropriate indices to determine the driving forces for their changes over time and across borders. Decomposition analysis is an important tool for quantifying and understanding the driving forces that underlie the changes in an economic, environmental, or energy-related indicator [50]. Index decomposition analysis (IDA) is a widely used method for decomposing changes in indicators at the sector level, and it can be used to detect the factors driving carbon emissions [51], historical carbon intensity [52], and the embodied carbon in trade [36, 53]. This approach can also be applied at a regional or city level [54]. IDA uses only aggregate sector information and was chosen for the current study due to its clear and policy-relevant interpretation of results.

In this study, temporal and spatial IDAs were adopted to decompose the driving forces of EEE and BEET, respectively. The temporal IDA illustrates how an indicator (EEE in the current study) is driven by domestic trade-related environmental and economic factors to change over time [27], and the spatial IDA can be used to illustrate how the differences in the trade-related environmental and economic characteristics between countries or regions (China and its trade partners in the current study) are related to the BEET and its variation [34, 35]. To highlight the geographical distribution of China’s major trade partners, the TEIs of European Union (EU), the Association of Southeast Asian Nations (ASEAN), the USA, Japan, Korea, Australia, Russia, and Taiwan were calculated from their respective SREIOs, and the TEIs of the rest of the world (ROW) were set to equal to China.

The Logarithmic Mean Divisia Index (LMDI) is the algorithmic method used to solve the IDA problem. This index has several useful features, including the avoidance of a residual term, yielding complete decomposition, and its ability to handle computational problems associated with zero values in the data set. A practical guide for the use of the LMDI approach was subsequently provided by Ang [55].

The IDA has three indicator forms: absolute, intensity, and elasticity [56]. In the temporal IDA, changes in the EEE are attributed to the ‘scale effect’, the ‘composition effect’ and the ‘technique effect’ [57], the last of which is attributed to the sum of ‘production efficiency effect’ and the ‘regulation effect’. In this study, the scale and composition effects indicate the changes in emissions caused by the scale and structure adjustment in the exports and the associated changes in production. The technique effect denoted by the total emissions intensity (TEI) for various production sectors reflects the reduction in emissions caused by improvements in the production efficiency (i.e., the production efficiency effect) and environmental regulation implementation (i.e., the regulation effect), or more specifically, energy savings and end-of-pipe reduction measures. The production efficiency effect and the regulation effect combine to shape the changes in the TEIs of each economic sector.

For EEE, the temporal IDA identity can be written as

$$EEE=\sum_{i}X\times \frac{x_{i}}{X}\times F_{i}=\sum_{i}X\times S_{i}\times F_{i},$$ (7)

where *X* is the total amount (in value) of the exports, which indicates the scale effect, *x*_*i*_ is the amount of exports of sector *i*, so *S*_i_ is the share of sector *i* of the total exports, which indicates the composition effect, and *F*_*i*_ is the total emissions intensity (TEI) of sector *i*.

Next,

$$F_{i}=\frac{TE_{i}}{O_{i}}=\frac{F_{i}\times O_{i}}{I_{i}}\times \frac{I_{i}}{O_{i}}=F_{i}\times TFP\times 1/TFP,$$ (8)

where *TE*_*i*_ is the total emissions of sector *i*, *O*_*i*_ is the total output of sector *i*, *I*_*i*_ is the total input of production factors, *TFP* (*O*_*i*_*/I*_*i*_) is the total factor productivity, or the ratio of the total output over the total input of the production factors, $\frac{F_{i}\times O_{i}}{I_{i}}$ (or *F*_*i*_ × *TFP*) is the pollutant emissions per unit total input of the production factors, which implies the regulation effect, and $\frac{1}{TFP}$ indicates the production efficiency effect. Formula (9) can be obtained from formulas (7) and (8):

$$EEE=\sum_{i}X\times S_{i}\times F_{i}=\sum_{i}X\times S_{i}\times \left(F_{i}\times TFP\right)\times \frac{1}{TFP}.$$ (9)

The additive mathematical model is employed to decompose the aggregate difference of total EEE for the period 0 to T:

$$EEE_{tot}^{T-0}=EEE^{T}-EEE^{0}=\Delta EEE_{scl}^{T-0}+\Delta EEE_{comp}^{T-0}+\Delta EEE_{reg}^{T-0}+\Delta EEE_{eff}^{T-0}.$$ (10)

The subscripts *scl*, *comp*, *reg* and *eff* denote the effects associated with the overall scale (total export), composition, regulation and the production efficiency, respectively. Note that the technique effect is set to be the sum of the regulation effect and production efficiency effect.

Next, the BEET (the difference between EEE and the EEI) was decomposed into three driving force factors via the spatial IDA. The ‘emission intensity effect **(**ΔEI)’ reflects how much the difference in the economy-wide emissions intensity (total emissions/GDP) between China and its trade partners (the world other than China) contributes to the formation of the BEET. The ‘specialization effect **(**ΔSP)’ reflects how much the difference in the degree of specialization in pollution-intensive products between China’s exports and imports contributes to the BEET. The ‘trade balance effect **(**ΔTB)’ reflects how much the difference between the exports and the imports contributes to the BEET. Our approach is based on Jakob and Marschinski and Gasim [34, 35].

In the spatial IDA, China’s EEE and EEI are decomposed into three factors in a slightly different way:

$$EEE=\frac{E_{c}}{GDP_{c}}\times \frac{\frac{EEE}{X}}{\frac{E_{c}}{GDP_{c}}}\times X=EI_{c}\times sp_{c}\times X,$$ (11)

$$EEI=\frac{E_{tp}}{GDP_{tp}}\times \frac{\frac{EEI}{M}}{\frac{E_{tp}}{GDP_{tp}}}\times M=EI_{tp}\times sp_{tp}\times M,$$ (12)

where subscripts c and tp denote China and its trade partners, respectively. E denotes the total emissions. GDP is the gross domestic product, EI (or E/GDP) is the economy-wide emissions intensity, and sp is the degree of “specialization in pollution-intensive products” of exports or imports. Specialization in pollution-intensive products expresses the ratio of the embodied GHGs or pollutant emissions in unit exports and imports over the averagely embodied emissions calculated from the GDPs of China and its trade partners following the approach of Leamer [58].

The BEET can then be decomposed into an economy-wide emissions intensity effect (ΔEI), a specialization in pollution-intensive products effect (ΔSP), and a trade balance effect (ΔTB):

$$\text{B}EET=EEE-EEI=EI_{c}\times sp_{c}\times X-EI_{tp}\times sp_{tp}\times M=\Delta EI+\Delta SP+\Delta TB,$$ (13)

Based on LMDI, the relevant formulas for the decomposition factors are listed in **S1 Text** to avoid unnecessary details.

IDA method is notably different from the typical statistical or econometric methods that micro-economists are familiar with. The IDA method with a LMDI solution has its merit that, it deliberately avoids error-based statistical analysis, and thus in the previous literature, it was referred to as an ideal decomposition method "not leaving a residual term" [59–61]. It should be noted that for the IDA analysis, the relationship between the dependent variable, for example, EEE and the independent variables, for example, total export, sectoral export, and sectoral TEI, are established, or in other words, the dependent variable is essentially calculated from the product of the independent variables; thus, the dependent variable is fully explained by all of the components. This decomposition method is different than statistical and econometrics methods, which essentially deal with the causal relationship between a dependent variable and its many independent explanatory variables, which are selected largely based on the researchers’ rational assumptions and hypothesis [62]. Although early IDA solutions such as the basic Laspeyres and the simple average Divisia methods [59, 63–64] left a residual, when an ‘appropriate’ solution method is applied for IDA, for example, the LMDI method used in the present study, a calculative residual could be avoided. A proof process in the **S2 Text** elaborates on this issue.

### 2.2 Data sources and data aggregation

China overtook the USA to become the world’s largest emitter of CO_2_ in 2007 [65], and CH_4_ and N_2_O are two other important greenhouse gases. China was the biggest source of SO_x_ in the world, and the problem of NO_x_ pollution was gaining increasing attention in China. The current study thus employed CO_2_, CH_4_, N_2_O, SO_x_ and NO_x_ as the emissions indicators for this study. CH_4_ and N_2_O were converted to CO_2_ equivalents (CO_2_-eq) according to the global warming potential (GWP) [66].

This paper estimates the yearly emissions from 2002–2011 based on input-output (I-O) tables developed from the World Input-Output Database (WIOD) [37]. The 35 sectors of the WIOD database were aggregated into 15 sectors for the convenience of this study, which are shown in **S1 Table**.

The current study adopted the sectoral pollutant emissions factor data and the I-O tables of China from the WIOD to develop the sectoral TEI matrix for China (*F*_*ex*_). Similar method was applied to China’s major trade partners, including the EU, ASEAN, the USA, Japan, Korea, Australia, Russia, and Taiwan (they in sum accounted for 63.3%-79.4% of the total imports of China during 2002–2011) and the rest of the world (ROW), to estimate the collective sectoral TEI matrix for China’s trade partners (*F*_*tp*_):

$$F_{tp}=\sum_{j}\alpha_{j}\times F_{j},$$ (14)

where *α*_j_ is the proportion (or weight) of the goods imported from trade partner *j* in China's total imports, and *F*_*j*_ represents the sectoral TEI matrix of the trade partner. The TEIs of the ROW, which are not available, were assumed to be equal to those of China. The sectoral TEIs of China and its trade partners utilized in this study are listed in **S3 Text**.

The study adopted the GDP data from the World Development Indicators (WDI) [1]. All monetary units were converted to 2005 US$ currency values based on the producer price index (PPI) of the USA [67].

## 3. Results and discussion

### 3.1 GHGs and pollutant emissions embodied in China’s international trade

**Fig 1:**
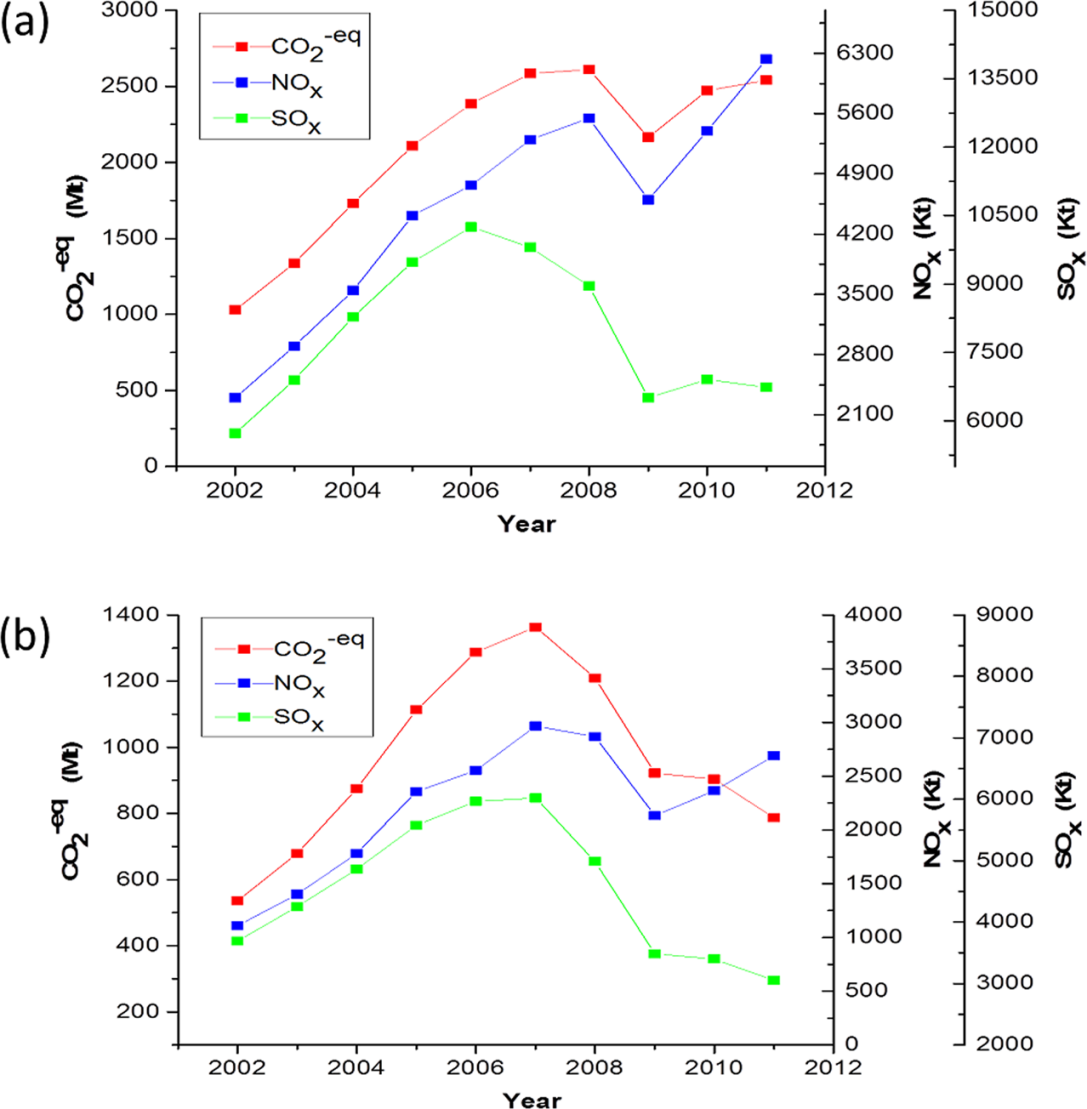
Environmental performance of China’s international trade. (a) Pollutant emissions embodied in China’s exports (EEE); (b) Balance of pollutant emissions embodied in China’s trade (BEET).

The EEEs and BEETs of China from 2002 to 2011 are shown in **Fig 1(A)** and **1(B)**. The EEEs of the GHGs and NO_x_ presented similar N-shaped trends, but that of SO_x_ suggested an inverted U shape. The EEEs of the GHGs and NO_x_ peaked in 2008, compared with SO_x_ which peaked in 2006. The EEEs of the GHGs and NO_x_ primarily decreased with the global financial crisis but increased again when the global economy and international trade revived from 2009 to 2011 [31]. Additionally, the EEE of SO_x_ started to decrease two years earlier than those of the other pollutants and remained relatively low thanks to the stricter end-of-pipe sulfur scrubber equipment requirement instituted in China’s 11^th^ FYP period (2006–2010) [68].

The EEEs of the GHGs, SO_x_ and NO_x_ from 2002–2011 respectively accounted for 28.96%, 32.01% and 25.63% of China’s total 10-year accumulated emissions. This result demonstrates the great contribution of foreign trade to total national emissions. It can be intuitively speculated that the reasons for the magnitude of the EEE were the huge export trade volume and the high emission intensity of China, which is explained in section 3.2.1.

China’s BEETs for the GHGs and SO_x_ presented inverted U-shaped trends, but NO_x_ exhibited an N-shaped trend. The maximum BEET values for 2007 are shown in **Fig 1(B)**. The accumulated BEETs for the GHGs, SO_x_ and NO_x_ from 2002 to 2011 respectively amounted to 14,225.9 megatons (Mt) (CO_2_-eq), 51,794.2 kilotons (kt) and 26,551.3 kt, and accounted for 19.66%, 20.84% and 15.13% of China’s total emissions. The positive BEETs meant that China was retaining a large surplus balance of pollutant emissions from international trade which, in fact, were even larger than the total pollutant emissions of the United Kingdom plus France of the same period. The magnitude of BEET might be attributed to the difference between the total emission intensity and the net trade balance, which is explained in section 3.2.2.

The EEEs and BEETs for China calculated by this study and in previous studies are listed in **S2 Table** for comparison. Our estimates of the proportion of the EEE and BEET for GHGs in the total annual emissions of China are basically within the upper and lower bounds provided by the literature [9, 12, 20–21, 23–24, 32, 44, 69–70].

### 3.2 Driving force analysis for EEE and BEET

#### 3.2.1 Temporal IDA for EEE

The EEE decomposition results are shown in Table 1. From 2002 to 2011, the scale effect played a major role in the increase of the accumulated total embodied GHGs emissions (3,459.6 Mt, CO_2_-eq). The technique effect (i.e., the production efficiency effect plus the regulation effect) decreased the accumulated total embodied GHGs emissions by 1,946.0 Mt (CO_2_-eq) from 2002 to 2011, to which the production efficiency effect contributed 362.1 Mt (CO_2_-eq) and the regulation effect contributed 1,583.9 Mt (CO_2_-eq). However, the composition effect played an insignificant role in the accumulated total embodied GHGs emissions (-3.4 Mt, CO_2_-eq).

**Table 1:** Decomposition results of pollutants embodied in China’s exports, 2002–2011.

Year	03–02	04–03	05–04	06–05	07–06	08–07	09–08	10–09	11–10	Total
GHGs (Mt, CO_2_-eq)										
Δ*EEE*_*tot*_	307.3	392.7	378.5	276.6	200.7	23.3	-444.8	308.4	67.3	1510.0
Δ*EEE*_*scl*_	333.4	459.6	465.0	533.4	585.0	418.8	-404.0	632.1	436.2	3459.6
Δ*EEE*_*comp*_	-10.3	3.0	8.6	-5.0	-7.4	38.7	-67.7	7.1	29.3	-3.5
Δ*EEE*_*tch*_	-15.8	-70.0	-95.1	-251.8	-376.9	-434.2	26.9	-330.9	-398.2	-1946.1
Δ*EEE*_*eff*_	-58.0	-28.5	-8.8	-49.1	185.9	-231.5	298.5	-452.1	-18.6	-362.1
Δ*EEE*_*reg*_	42.2	-41.5	-86.3	-202.8	-562.9	-202.7	-271.6	121.2	-379.6	-1583.9
NO_x_ (Kt)										
Δ*EEE*_*tot*_	601.2	645.2	869.7	351.9	531.4	250.7	-951.9	802.3	832.5	3933.0
Δ*EEE*_*scl*_	733.1	968.6	963.7	1090.9	1184.1	874.6	-859.1	1361.8	1010.4	7328.0
Δ*EEE*_*comp*_	-17.8	16.9	14.9	2.2	-5.6	51.1	-80.2	12.3	48.8	42.5
Δ*EEE*_*tch*_	-114.1	-340.3	-108.9	-741.2	-647.0	-675.0	-12.6	-571.8	-226.7	-3437.6
Δ*EEE*_*eff*_	-127.5	-60.0	-18.3	-100.3	376.3	-483.4	634.8	-973.9	-43.1	-795.5
Δ*EEE*_*reg*_	13.4	-280.4	-90.6	-640.8	-1023.3	-191.5	-647.4	402.2	-183.5	-2642.0
SO_x_ (Kt)										
Δ*EEE*_*tot*_	1177.5	1379.3	1201.0	770.1	-441.5	-857.7	-2443.1	401.6	-172.4	1014.7
Δ*EEE*_*scl*_	1783.0	2281.8	2155.6	2343.3	2360.6	1511.8	-1300.7	1831.3	1187.4	14154.1
Δ*EEE*_*comp*_	11.2	178.7	76.7	98.7	44.3	147.1	-295.7	42.4	88.1	391.4
Δ*EEE*_*tch*_	-616.8	-1081.2	-1031.3	-1671.8	-2846.4	-2516.6	-846.7	-1472.1	-1447.9	-13530.8
Δ*EEE*_*eff*_	-310.1	-141.3	-40.9	-215.5	750.2	-835.7	961.1	-1309.7	-50.7	-1192.6
Δ*EEE*_*reg*_	-306.6	-939.9	-990.4	-1456.3	-3596.5	-1681.0	-1807.8	-162.4	-1397.2	-12338.2

Between 2002 and 2008, the scale effect led to most of the increase in the accumulated total embodied GHGs emissions (2,795.2 Mt, CO_2_-eq), and the technique effect mitigated the accumulated total embodied emissions by 1,243.8 Mt (CO_2_-eq). However, between 2008 and 2009, the scale effect caused a 403.9 Mt (CO_2_-eq) decrease, thanks to the world financial crisis which caused a shrinkage in world trade volume. And then the scale effect returned to be the effective driver of the growth of EEE. The technique effect caused an annual average 294.2 Mt (CO_2_-eq) reduction from 2005 to 2011 in the export embodied emissions compared to the annual average 60.3 Mt (CO_2_-eq) reduction from 2002 to 2005, largely because of stricter energy-saving and carbon-reduction measures. Similar trends and driving force decomposition results observed for the GHGs also occurred for SO_x_ and NO_x_.

The technique effect is reflected by the changes in the TEIs of the various sectors. The TEIs of all 15 sectors from 2002 to 2011 were calculated using an EIO model. From 2002 to 2011, the TEI across sectors for every pollutant declined by at least 40%. The highest rate of decline was observed for SO_x_ (85.87%), followed by N_2_O (60.76%), CH_4_ (60.17%), CO_2_ (57.14%) and NO_x_ (48.95%). Within the technique effect, the regulation effect played a more important role than the production efficiency effect, which nonetheless improved; in other words, the end-of-pipe reduction measures of the 11^th^ FYP were the main reason for the emissions intensity decrease. The technique effect observed for NO_x_ was considerably smaller than that for SO_x_ because NO_x_ was not among the pollutants targeted for reduction as part of the 11^th^ FYP. Therefore, the EEE of NO_x_ grew rapidly after the global financial crisis.

Although China's trade structure has recently changed to a certain extent, the composition effect did not lead to a notable change in the EEE. This is largely because the sectors whose exports increased and the sectors whose exports decreased have similar TEIs; the increase and decrease in emissions basically almost cancelled each other out when taking all of the sectors as a whole. The share of the contribution of high-polluting, high-energy-consuming and high-emitting sectors to total exports remains largely unchanged, such as the sectors of mining (MIN), non-metal mineral products (NMP) and metals and metal products (MMP).

#### 3.2.2 Spatial IDA for BEET

**Fig 2:**
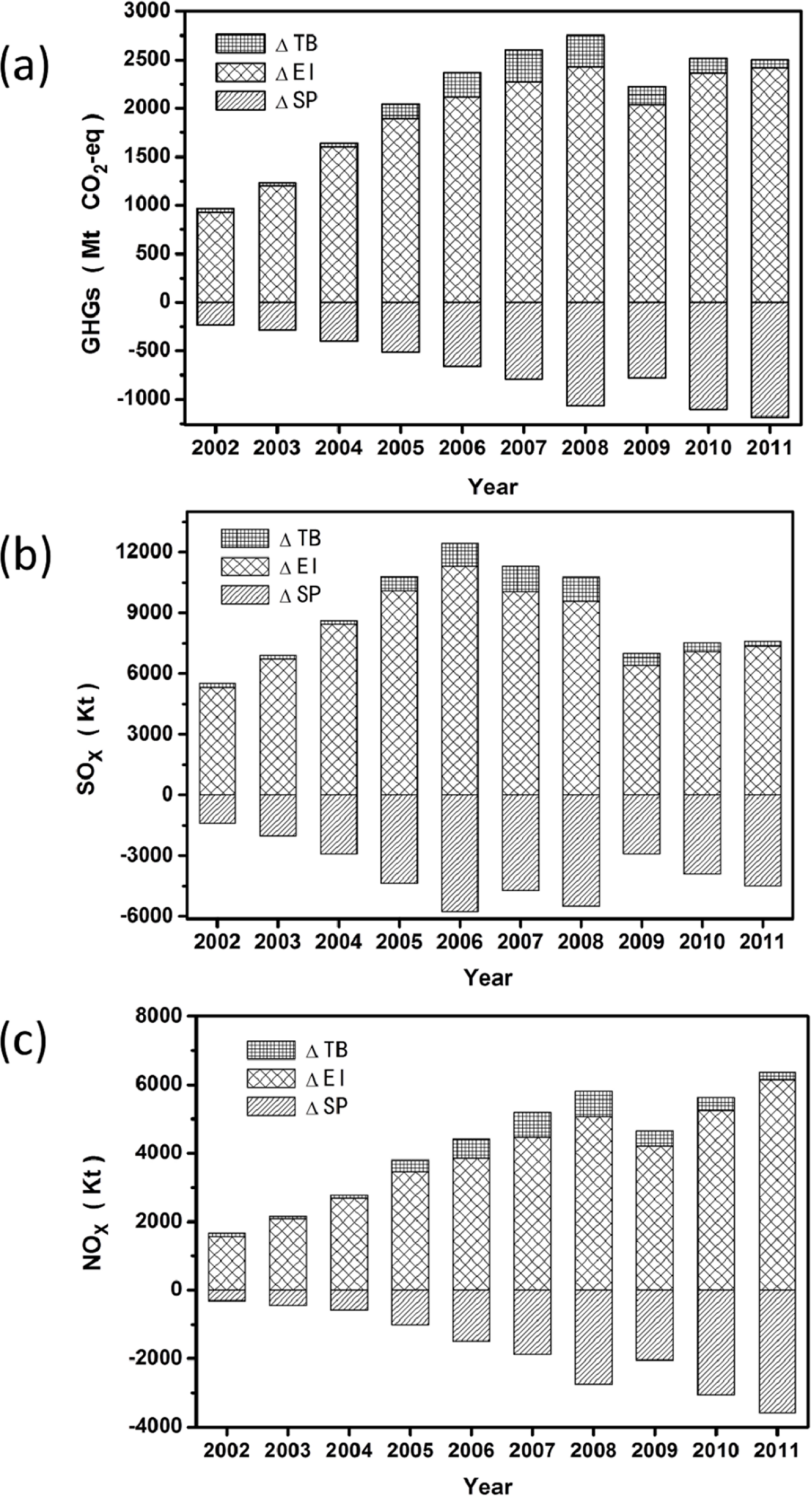
Decomposition of pollutants embodied in China’s net exports (BEET), 2002–2011. **(a) GHGs; (b) SO**_**x**_**; (c) NO**_**x**_. Note: data of this figure are listed in **S3 Table.**

Unlike the decomposition analysis for EEE—whose basis and change basically depend on internal factors such as the export volume, the production efficiency, regulation, and emissions intensity within China—the basis and change of BEET is driven by the differences of China and its trade partners, in the economy-wide emission intensities of the goods production (EI), the degree of specialization in producing pollution intensified goods (sp), and the trade surplus (the ratio of X/M was used to indicate the degree of difference in export and import). Thus, the BEET is factored into the intensity effect (ΔEI), the specialization effect (ΔSP), and the trade balance effect (ΔTB). (see Fig 2(A)–2(C) for the decomposition results for different pollutants)

**Fig 3:**
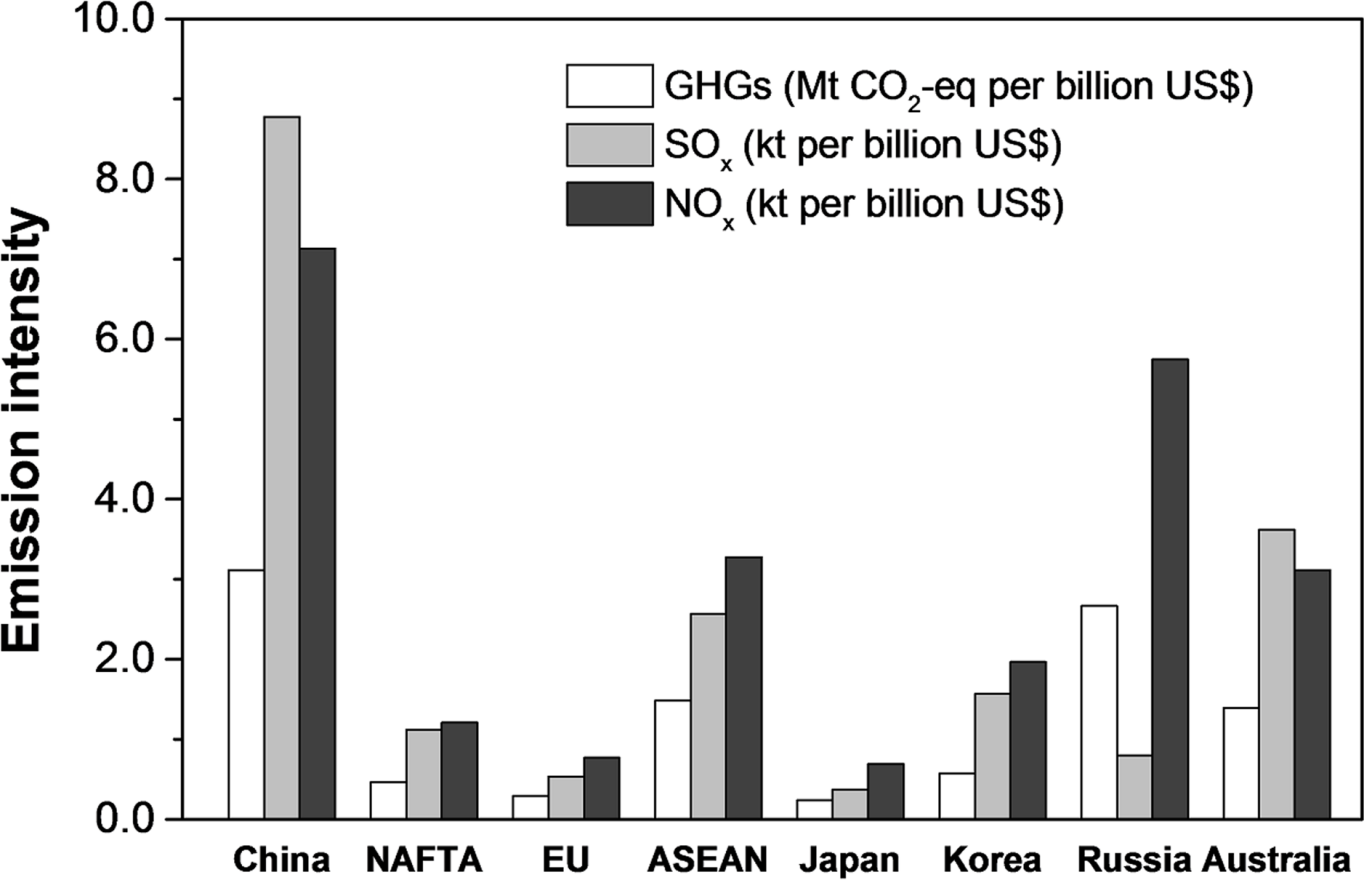
The average emission intensities of China and its major trade partners (2002–2011).

Although China’s economy-wide emissions intensity (EI_c_) declined steeply in recent years, a large gap still existed when compared with those of developed countries and regions. The aggregated average EIs of China’s major trade partners (mostly developed countries and regions) are more than 60% lower than those of China. The intensity effect (ΔEI) has been maintained at a high level from 2002 to 2011 and played the most important role in the basis of the BEET. (see **Fig 3**)

**Fig 4:**
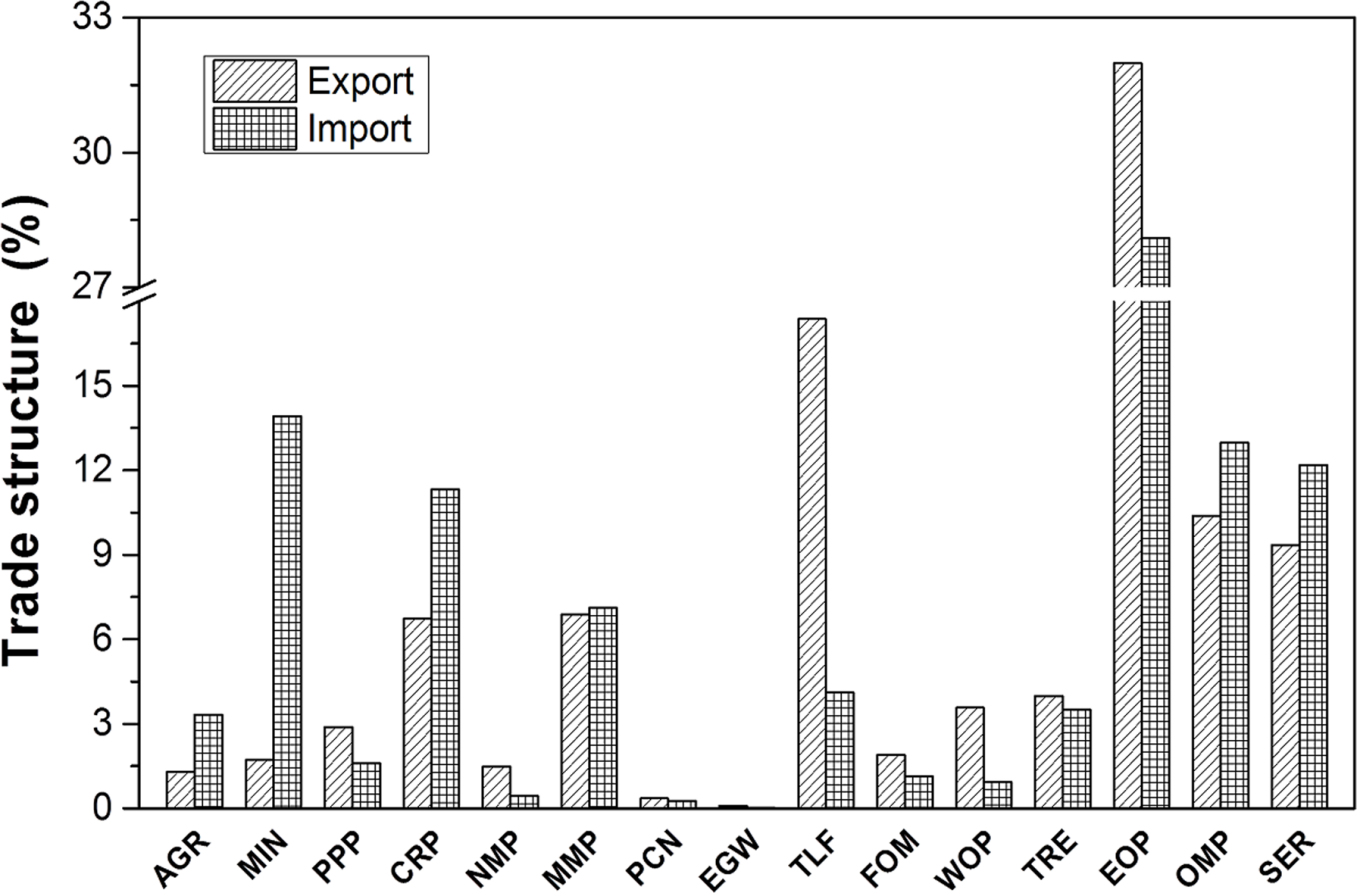
Comparison of the proportion of average export and import value for various sectors in China (2002–2011). Note: abbreviations of the sectors are listed in **S1 Table**.

Mining (MIN), paper and publishing products (PPP), chemicals (CRP), non-metal mineral products (NMP) and metals and metal products (MMP) are generally regarded as “dirty sectors” with larger TEIs (either in China or its trade partners). It was found that the dirty sectors accounted for 18.31% to 20.61% of China’s exports, and in terms of imports, the dirty sectors accounted for 30.46% to 39.08%. (see **Fig 4**) This finding confirmed that China had a higher degree of specialization (sp) in pollution-intensive products in its imports than in its exports, namely, sp_tp_ > sp_c_, which resulted in a negative specialization effect (ΔSP) that offset a portion of the enormous positive ΔEI.

China’s trade surplus increased from US$ 25.4 billion in 2002 to US$ 290.5 billion in 2008, which contributed to the sustained growth in the BEET from 2002, and it remained at a high level until 2008. However, after 2009, China’s trade surplus declined significantly, decreased to US$ 105.6 billion in 2011, and the trade balance effect (ΔTB) also consequently declined, but it did not play an important role in the basis of BEET.

**S4 Table** presents the yearly ratios of EI_c_/EI_tp_, sp_c_/sp_tp_ and X/M from 2002 to 2011. The economy-wide emissions intensity in China and its trade partners (EI_c_ and EI_tp_) continued to decrease from 2002 to 2011 for all GHGs, SO_x_ and NO_x_. However, the emission intensities of GHGs and SO_x_ in China decreased at a lower rate when compared with the level in the rest of the world during the 10^th^ FYP period; however, the decrease accelerated as the 11^th^ FYP period began. Thus, the EI_c_/EI_tp_ for the GHGs and SO_x_ increased during the 10^th^ FYP period and the trend was reversed in the 11^th^ FYP period. Yet, the EI_c_/EI_tp_ for NO_x_ continued to increase throughout the 10^th^ and 11^th^ FYP periods. These facts reconfirmed the effectiveness of stricter energy-saving measures and the SO_2_ emission reduction measures that had been implemented, but little attention was given to NO_x_ control in the 10^th^ and 11^th^ FYP periods [71–72].

Specialization of the export in the pollution-intensive goods (sp_c_) for all three pollutants increased during the 10^th^ FYP period and then decreased in the 11^th^ FYP period, indicating a slight change in favor of cleaner exports. At the same time, the sp_tp_ for the GHGs and SO_x_ experienced a change similar to sp_c_, and that for NO_x_ continued to increase slightly. The ratios of sp_c_/sp_tp_ <1 for the GHGs, SO_x_ and NO_x_ generally decreased annually, indicating that compared with its imports, China’s exports tended to be preponderantly cleaner products to a certain extent in recent years.

### 3.3 Policy discussion

The composition effect played an insignificant role on the variation of EEEs according to the temporal IDA results, which means the structure of China’s export had not been improved enough from the perspective of environment protection. The problem of the mix of export also reflects and discloses the urgency of supply-side structure transformation in China. On the one hand, for the long period, it has been criticized that the proportion of the industries and products with low value-added, high energy consumption and high pollutants emissions was excessively high, which could be driven down by the improved export structure to be cleaner and more environmentally friendly. On the other hand, China’s great efforts to promote “the supply-side structure reform” through resolving the overcapacity of steel, plate glass, cement, chemicals among others, are expected to help to upgrade the export mix and reduce EEEs. Although the decrease of sectoral TEIs has offset a large part of the increase of EEEs, currently the room for energy saving and emissions reduction is still large. The government should further accelerate the TEIs decrease through enforcement of environmental regulation and standards and encourage innovation-driven development.

According to the “National New-Type Urbanization Plan (2014–2020)”, by the year 2020, 60% of the population in China will be living in urbanized areas, which means more than 100 million rural people will be relocated to the urban areas. The per capita consumption of urban people and rural people was calculated using the China Statistical Yearbook and input-output tables (2010 and 2012) of China [73–76]. Based on China’s IO table and the TEIs calculated in this study, the impact of the urbanization of 100 million rural people can be obtained. The urbanization policy could increase the annual household consumption (estimation based on 2011 level) by 7.76% because urban people consume more than rural people, and in particular, they spend more on services. The increment of annual emissions will be 329.74 Mt CO2-eq for the GHGs, 681.09 kt for SO_x_ and 705.56 kt for NO_x_, respectively accounting for 3.41%, 2.88% and 2.78% of China’s total emissions. Though the percentage changes of emissions are small, the impacts should not be neglected because those emissions (specially the SO_x_ and NO_x_) are mostly concentrated in high population density areas. To curb the emission increase, a more aggressive import policy for intermediates and final goods especially those generating high pollutant emissions, should be implemented to reduce domestic and global emissions.

Although a more aggressive import policy is optional for rapidly developing countries such as China, cross-border technology transfer would be more desirable to help to reduce global emissions and at the same time enable the people to benefit from freer trade. The international division of production and the transfer of polluting production from developed to developing countries and regions is a natural process according to international trade theories because developing countries and regions have the comparative advantage of a cheaper labor forces and natural resources. However, gaps exist between the developed and developing trade partners in the economy-wide emissions intensity and the sectoral TEIs. These gaps had led to an embarrassing situation: while the GHGs and traditional pollutant emissions of the developed trade partners had decreased, the emissions of the developing trade partners were increasing at a magnitude much larger than the reductions in the former [18, 70]. This de facto defect of modern international trade gives rise to the strong and robust argument that the transfer of advanced production technologies and emission control technologies from developed to developing countries should be a compulsory environmental policy option. Thus, the world as a whole is able to utilize the cheap and abundant labor force (and other resources) to the advantage of developing countries, and at the same time keep the global emissions of GHGs and other pollutants low.

### 3.4 Uncertainty analysis

Although the current study strove to optimize the analysis, it is necessary to be aware of the uncertainties of the raw and processed data. First, the raw data for the present study were drawn mainly from the WIOD database, which was based on official and publicly available data from statistical institutes to ensure a high level of data quality. In particular, the WIOD database was constructed within the framework of the international System of National Accounts and obeys its concepts and accounting identities, which restricted the number of countries that could be covered in WIOD because a trade-off exists between quality and coverage [77]. Second, the productions for processing exports and normal exports and relevant pollution emissions were not differentiated. The traditional I–O model uses the uniform export assumption for processing and normal exports. However, the intermediate inputs for processing exports are mainly imported from abroad, while those for normal exports are mainly from the domestic supply. The estimated CO_2_ emissions embodied in China's exports in the year 1997 can decrease by a quarter when using a processing export extended I-O model [42]. Third, the role of the composition effect might have not been fully reflected in this study. If the decomposition was conducted at a more disaggregated or sectoral level, structural changes might have been found to play a different role in the embodied emissions [27]. Lastly, when calculating the BEETs, the weighted average TEIs of China’s major trade partners were adopted to estimate the total emissions embodied in China’s imported goods; however, the TEIs of the ROW, which were not available, were assumed to be equal to those of China, which could lead to deviations in the EEI and BEET estimates.

The uncertainty analysis of decomposition results are shown in **S4 Text**. The decomposition results are not greatly affected by 5% perturbations of the data and thus the robustness of the decomposition results is confirmed.

## 4. Conclusions

By quantifying the embodied GHGs and pollutant emissions in China’s trade from 2002 to 2011, it was confirmed that the production of exports had a significant environmental impact on China. The EEEs accounted for approximately 30% of total emissions (GHGs, SO_x_ and NO_x_) in China. However, China has effectively reduced the emissions embodied in its exports by reducing the emissions intensity (reflected by the sectoral TEIs), and the financial crisis of 2008 to 2009 also ameliorated the emissions pressure. The positive BEETs for the GHGs, SO_x_ and NO_x_ (respectively accounting for 19.66%, 20.84% and 15.13% of China’s total emissions) means that China has been retaining a large surplus of pollutant emissions from international trade with a volume even larger than the total emissions of the United Kingdom plus those of France.

From a domestic point of view, the temporal decomposition analysis revealed that the growth of exports (a scale effect) had a large influence on the growth of the embodied emissions during 2002–2007. Although the scale effect dominated, the technique effect (a combination of the production efficiency and regulation effects) offset the embodied emissions to a large extent from 2007 to 2011. Stricter domestic emission control policies had been the primary measures to restrain the SO_x_ emissions embodied in the exports after 2006. The composition effect played an insignificant role during this process.

The spatial decomposition analysis revealed how the comparative differences between China and its major trade partners drove the basis for and the changes in the BEETs. Thus, the BEETs were attributed to the intensity effect (ΔEI), the specialization effect (ΔSP) and the trade balance effect (ΔTB). Although China’s EI declined steeply, a large gap still existed when it was compared with its major trade partners (mostly developed countries and regions), which maintains China in an environmentally inferior position. It was confirmed that China had a higher degree of specialization in pollution-intensive products in its imports than in exports which, to a certain extent, offset the huge positive emissions intensity effect. Researchers have argued that an excessive trade surplus has not only complicated China’s balance of international payments, but also led to unbalanced emissions embodied in trade [68]. The findings of this study support this argument, however it was found that the trade surplus did not play an important role in the basis of the BEET compared with the emissions intensity effect.

Exports form part of the troika of China's economic growth (the other two components are investment and domestic consumption). When China continues pushing for trade liberalization in the future, policy measures must be implemented to reduce adverse environmental effects. According to the findings of this study, the scale effect or the magnitude of exports and the economy-wide high emissions intensity, are the major contributors to excess emissions, which can have a negative environmental impact. China should take effective measures to hold its irrational motivation to expand exports and at the same time optimize export structure and reduce the total emissions intensity. A more aggressive import policy was useful for curbing domestic and global emissions, and the cross-border transfer of advanced production technologies and emission control technologies from developed to developing countries should be a compulsory global environmental policy to mitigate the leakage of pollution emissions caused by international trade.

## Supporting information

S1 Text The decomposition formulas of LMDI method. (DOCX)

S2 Text Introduction to the index decomposition analysis (IDA) and Logarithmic Mean Divisia Index (LMDI) method. (DOCX)

S3 Text China’s and its major trade partners’ total emissions intensity (2002–2011). (DOCX)

S4 Text Uncertainty analysis of decomposition results. (DOCX)

S1 Table Sector classification for the current study. (DOCX)

S2 Table A Comparison of EEEs and BEETs of China in this study with previous studies. Note: * Average value of the calculation years.
(DOCX)

S3 Table Decomposition of pollutants embodied in China’s net exports (BEET), 2002–2011. (DOCX)

S4 Table Yearly ratio of EI_c_/EI_tp_, sp_c_/sp_tp_ and X/M during 2002–2011. Note: EI_c_/EI_tp_ is the economy-wide emissions intensity of China divided by its trade partners; sp_c_/sp_tp_ is the degree of ‘pollution intensive product specialization’ of exports of China divided by that of its trade partners.
(DOCX)
