# Supplementary material for: GHGs and air pollutants embodied in China’s international trade: Temporal and spatial index decomposition analysis
Source: PLoS One. 2017 Apr 25;12(4):e0176089. doi: 10.1371/journal.pone.0176089 (PMC5404823; doi:10.1371/journal.pone.0176089)
Supplement: S1 Text — (DOCX) [file pone.0176089.s001.docx]

## S1 Text. The decomposition formulas of LMDI method

The specific LMDI formulas for temporal IDA for EEE are as follows:

${\Delta EEE}_{scl}^{T-0}=\sum_{i} \frac{{EEE}_{i}^{T}-{EEE}_{i}^{0}}{\ln{EEE}_{i}^{T}-\ln{EEE}_{i}^{0}}\ln\left( \frac{X^{T}}{X^{0}} \right)$, (1)${\Delta EEE}_{comp}^{T-0}=\sum_{i} \frac{{EEE}_{i}^{T}-{EEE}_{i}^{0}}{\ln{EEE}_{i}^{T}-\ln{EEE}_{i}^{0}}\ln\left( \frac{S_{i}^{T}}{S_{i}^{0}} \right),$ (2)

${\Delta EEE}_{reg}^{T-0}=\sum_{i} \frac{{EEE}_{i}^{T}-{EEE}_{i}^{0}}{\ln{EEE}_{i}^{T}-\ln{EEE}_{i}^{0}}\ln\left( \frac{F_{i}^{T}{TFP}^{T}}{F_{i}^{0}{TFP}^{0}} \right),$ (3)

${\Delta EEE}_{eff}^{T-0}=\sum_{i} \frac{{EEE}_{i}^{T}-{EEE}_{i}^{0}}{\ln{EEE}_{i}^{T}-\ln{EEE}_{i}^{0}}\ln\left( \frac{\frac{1}{{TFP}^{T}}}{\frac{1}{{TFP}^{0}}} \right)$. (4)

the specific LMDI formulas for spatial IDA for BEET are as follows:

$\Delta IE=\frac{EEE-EEI}{\ln EEE-\ln EEI}\times ln( \frac{{EI}_{c}}{{EI}_{tp}})$, (5)

$\Delta SP=\frac{EEE-EEI}{\ln EEE-\ln EEI}\times ln( \frac{{sp}_{c}}{{sp}_{tp}})$, (6)

$\Delta TB=\frac{EEE-EEI}{\ln EEE-\ln EEI}\times ln( \frac{X}{M}).$ (7)
