# Supplementary material for: GHGs and air pollutants embodied in China’s international trade: Temporal and spatial index decomposition analysis
Source: PLoS One. 2017 Apr 25;12(4):e0176089. doi: 10.1371/journal.pone.0176089 (PMC5404823; doi:10.1371/journal.pone.0176089)
Supplement: S2 Text — (DOCX) [file pone.0176089.s002.docx]

## S2 Text. Introduction to the index decomposition analysis (IDA) and Logarithmic Mean Divisia Index (LMDI) method

The decomposition methodology is a technique that provides a linkage between an aggregate and the original raw data whereby information of interest is captured in a concise and usable form. The concept is similar to the application of economic index numbers to the study of contributions of price and quantity levels to changes in aggregate commodity consumption. It is therefore appropriate to name the methodology index decomposition (ID) (index decomposition analysis used in subsequent research) [1].

IDA was used to deal with energy consumption since around 1980 to quantify drivers behind changes in an aggregate of energy consumption or the energy intensity [2-5]. The IDA methods could be grouped into four types, i.e. Laspeyres (LASP), Shapley/Sun (S/S), logarithmic mean Divisia index (LMDI), and other Divisia method (including the arithmetic mean Divisia method (AMDI) and other parametric Divisia methods [6]). There was a shift from the conventional Laspeyres method and other Divisia methods which leave a residual term in the decomposition results, to ideal decomposition methods such as LMDI and the S/S method and in particular LMDI [7]).

The emission embodied in export (EEE) is expressed in the current study as:

$EEE=\sum_{i} X\times S_{i}\times F_{i}=\sum_{i} X\times S_{i}\times(F_{i}\times TFP)\times\frac{1}{TFP}$ (1)

where *X* is the total amount (in value) of exports, which indicates the scale effect; *S*_i_ is the share of sector *i* of the total exports, which indicates the composition effect; *F_i_* is the total emissions intensity (TEI) of sector *i*. *TFP* is the total factor productivity, $(F_{i}\times TFP)$ is the pollutant emissions per unit total input of production factors, which implies the regulation effect; and $\frac{1}{TFP}$ indicates the production efficiency effect.

Suppose the EEE varies from ${EEE}^{0}$ in time 0 to ${EEE}^{T}$ in time *T*. Such a change may be expressed in two ways:

${EEE}_{tot}^{T-0}=\frac{{EEE}^{T}}{{EEE}^{0}}=D_{scl}^{T-0}D_{comp}^{T-0}D_{reg}^{T-0}D_{eff}^{T-0}$ (2)

and

${EEE}_{tot}^{T-0}={EEE}^{T}-{EEE}^{0}=\Delta{EEE}_{scl}^{T-0}+\Delta{EEE}_{comp}^{T-0}+\Delta{EEE}_{reg}^{T-0}+\Delta{EEE}_{eff}^{T-0}$. (3)

We refer to the first as *multiplicative decomposition*, and the second way is called *additive decomposition* The subscripts scl, comp, reg and eff denote the effects associated with the overall scale (total export), composition, regulation and the production efficiency, respectively.

The Divisia index is an integral index number introduced by Divisia (1925) [8]. Hulten (1973) and Diewert (1980) further expound more details about this index number [9-10]. Applying the theorem of instantaneous growth rate to Eq. (S8) leads to:

$\frac{\mathrm{dln}\left( {EEE}^{T} \right)}{dt}=\sum_{i} w_{i}[\frac{dln\left( X \right)}{dt}+\frac{dln\left( S_{i} \right)}{dt}+\frac{dln\left( F_{i}\times TFP \right)}{dt}+dln(\frac{1}{TFP})/dt]$ (4)

where $w_{i}={EEE}_{i}^{T}/{EEE}^{T}$is the sector share of EEE and is known as the weight for sector *i* in the summation.

$ln(\frac{{EEE}^{T}}{{EEE}^{0}})=\int_{0}^{T} \sum_{i} w_{i}[\frac{dln\left( X \right)}{dt}]+\int_{0}^{T} \sum_{i} w_{i}\left[ \frac{dln\left( S_{i} \right)}{dt} \right]+\int_{o}^{T} \sum_{i} w_{i}[\frac{dln\left( F_{i}\times TFP \right)}{dt}]+\int_{0}^{T} \sum_{i} w_{i}[dln(\frac{1}{TFP})/dt]$ (5)

Eq. (S12) can be expressed in the multiplicative form Eq.(S9) where:

$D_{scl}^{T-0}=\exp\left[ \int_{0}^{T} \sum_{i} w_{i}[\frac{dln\left( X \right)}{dt}] \right]$ (6)

$D_{comp}^{T-0}=\exp\left[ \int_{0}^{T} \sum_{i} w_{i}\left[ \frac{dln\left( S_{i} \right)}{dt} \right] \right]$ (7)

$D_{reg}^{T-0}=\exp\left[ \int_{0}^{T} \sum_{i} w_{i}[\frac{dln\left( F_{i}\times TFP \right)}{dt}] \right]$ (8)

$D_{eff}^{T-0}=\exp\left[ \int_{0}^{T} \sum_{i} w_{i}[dln(\frac{1}{TFP})/dt] \right]$ (9)

Since only discrete data are available in empirical studies, the weight function is often approximated by the arithmetic mean of the weights for year 0 and year T which is called Simple Average Divisia index methods or Arithmetic Mean Divisia index method. As a result of this approximation, there is an existence of a residual.

Sato (1976) proposed the following weight function which gives the logarithmic mean of x and y in his search for an ideal log-change index formula [11]:

$L(x,y) = (y-x)/ln(y/x)$ (10)

It was defined that L(x,x) = x , which is the limit of L(x,y) as y🡪x. We also define the special case L(0,0) = 0. Tornqvist et al. (1985) specified that x and y must be positive numbers and L(x>y) has the range of (xy)^1/2^ < L(x,y) < (x+y)/2 when x is not equal to y. It can be seen that the weight function is symmetric, i.e., L(x,y) = L(y,x) [12].

Replacing x and y in Eq. (S17) by ${EEE}^{T}$ and ${EEE}^{0}$, respectively, yields

$L\left( {EEE}_{i}^{T}{,EEE}_{i}^{0} \right)=({EEE}_{i}^{T}-{,EEE}_{i}^{0})/ln(\frac{{EEE}_{i}^{T}}{{,EEE}_{i}^{0}})$ (11)

Sato (1976) independently found that this function could be used to discretize the integral Divisia index formulae to give an ideal index [11]. Using this weight scheme, Eqs. (S19) to (S22) can respectively be transformed into:

${\Delta EEE}_{scl}^{T-0}=\sum_{i} \frac{{EEE}_{i}^{T}-{EEE}_{i}^{0}}{\ln{EEE}_{i}^{T}-\ln{EEE}_{i}^{0}}\ln\left( \frac{X^{T}}{X^{0}} \right)$, (12)${\Delta EEE}_{comp}^{T-0}=\sum_{i} \frac{{EEE}_{i}^{T}-{EEE}_{i}^{0}}{\ln{EEE}_{i}^{T}-\ln{EEE}_{i}^{0}}\ln\left( \frac{S_{i}^{T}}{S_{i}^{0}} \right),$ (13)

${\Delta EEE}_{reg}^{T-0}=\sum_{i} \frac{{EEE}_{i}^{T}-{EEE}_{i}^{0}}{\ln{EEE}_{i}^{T}-\ln{EEE}_{i}^{0}}\ln\left( \frac{F_{i}^{T}{TFP}^{T}}{F_{i}^{0}{TFP}^{0}} \right),$ (14)

${\Delta EEE}_{eff}^{T-0}=\sum_{i} \frac{{EEE}_{i}^{T}-{EEE}_{i}^{0}}{\ln{EEE}_{i}^{T}-\ln{EEE}_{i}^{0}}\ln\left( \frac{\frac{1}{{TFP}^{T}}}{\frac{1}{{TFP}^{0}}} \right)$. (15)

This decomposition method is the Logarithmic Mean Divisia Index (LMDI) used in this study to solve the IDA problem; it has several useful features, including not leaving a residual term, yielding perfect decomposition, and its being able to handle computational problems associated with zero values in the data set.

It then can be proved that the LMDI method gives no residual through the following procedure [13]:

$\left( {EEE}^{T}-{EEE}^{0} \right)-\left[ \sum_{i} \frac{{EEE}_{i}^{T}-{EEE}_{i}^{0}}{\ln{EEE}_{i}^{T}-\ln{EEE}_{i}^{0}}\ln\left( \frac{X^{T}}{X^{0}} \right)+\sum_{i} \frac{{EEE}_{i}^{T}-{EEE}_{i}^{0}}{\ln{EEE}_{i}^{T}-\ln{EEE}_{i}^{0}}\ln\left( \frac{S_{i}^{T}}{S_{i}^{0}} \right)+\sum_{i} \frac{{EEE}_{i}^{T}-{EEE}_{i}^{0}}{\ln{EEE}_{i}^{T}-\ln{EEE}_{i}^{0}}\ln\left( \frac{F_{i}^{T}{TFP}^{T}}{F_{i}^{0}{TFP}^{0}} \right)+\sum_{i} \frac{{EEE}_{i}^{T}-{EEE}_{i}^{0}}{\ln{EEE}_{i}^{T}-\ln{EEE}_{i}^{0}}\ln\left( \frac{\frac{1}{{TFP}^{T}}}{\frac{1}{{TFP}^{0}}} \right) \right]=\left( {EEE}^{T}-{EEE}^{0} \right)-\left[ \sum_{i} \frac{{EEE}_{i}^{T}-{EEE}_{i}^{0}}{\ln{EEE}_{i}^{T}-\ln{EEE}_{i}^{0}}\ln\left( \frac{X^{T}S_{i}^{T}F_{i}^{T}{TFP}^{T}\frac{1}{{TFP}^{T}}}{X^{0}S_{i}^{0}F_{i}^{0}{TFP}^{0}\frac{1}{{TFP}^{0}}} \right) \right]=\left( {EEE}^{T}-{EEE}^{0} \right)-[\sum_{i} ({EEE}_{i}^{T}-{EEE}_{i}^{0})]$=0. (16)

**REFERENCE**

1. Ang BW, Zhang F (2003) A Survey of Index Decomposition Analysis in Energy and Environmental Studies, Energy 25: 1149–1176.
2. Boyd G, McDonald JF, Ross M, Hanson DA (1987) Separating the Changing Composition of US Manufacturing Production from Energy Efficiency Improvements: A Divisia Index Approach, Energy J. 8: 77-96.
3. Boyd G, Hanson DA, Sterner T (1988) Decomposition of Changes in Energy Intensity: A Comparison of the Divisia Index and Other Methods, Energ. Econ. 10: 309-312.
4. Reitler W, Rudolph M, Schaefer H (1987) Analysis of the Factors Influencing Energy Consumption in Industry: A Revised Method, Energ. Econ. 9:145-148.
5. Huntington HG (1989) The Impact of Sectoral Shifts in Industry on US Energy Demand, Energy 14: 363-372.
6. Ang BW (1995) Decomposition Methodology in Industrial Energy Demand Analysis, Energy 20:1081-1095.
7. Xu X, Ang BW (2013) Index Decomposition Analysis Applied to CO_2_ Emission Studies, Ecol. Econ. 93:313-329.
8. Division F (1925) Lidice Monetaire et al Theorie de la Monnaie, Revue Divisia Economic Politique 9: 109-135.
9. Hulten CR (1973) Divisia Index Numbers, Econometrica 41: 1017-1025.
10. Diewert WE (1980) Recent Developments in the Economic Theory of Index Numbers: Capital and the Theory of Productivity, Am. Econ. Rev. 70:260-267.
11. Sato K (1976) The Ideal Log-Change Index Number, Rev. Econ. Sta. 58: 223-228.
12. Tornqvist L, Vartia P, Vartia YO (1985) How should relative changes be measured? Am. Sta. 39: 43–46.
13. Ang BW, Zhang F, Choi K (1998) Factorizing Changes in Energy and Environmental Indicators through Decomposition, Energy 6: 489-495.
