# Supplementary material for: GHGs and air pollutants embodied in China’s international trade: Temporal and spatial index decomposition analysis
Source: PLoS One. 2017 Apr 25;12(4):e0176089. doi: 10.1371/journal.pone.0176089 (PMC5404823; doi:10.1371/journal.pone.0176089)
Supplement: S3 Text — (DOCX) [file pone.0176089.s003.docx]

**S3 Text. China’s and its major trade partners’ total emissions intensity (2002-2011).**

**Table A. China’s Total CO_2_ Emissions Intensity (2002-2011) (F_ex_ of CO_2_, Kt per US$ Million)**

|  | 2002 | 2003 | 2004 | 2005 | 2006 | 2007 | 2008 | 2009 | 2010 | 2011 |
| --- | --- | --- | --- | --- | --- | --- | --- | --- | --- | --- |
| AGR | 1.11 | 1.10 | 1.01 | 0.96 | 0.87 | 0.73 | 0.56 | 0.56 | 0.50 | 0.43 |
| MIN | 2.81 | 2.93 | 2.78 | 2.61 | 2.37 | 2.13 | 1.82 | 1.81 | 1.58 | 1.35 |
| FOM | 1.39 | 1.34 | 1.25 | 1.18 | 1.05 | 0.90 | 0.74 | 0.73 | 0.65 | 0.56 |
| TLF | 1.47 | 1.51 | 1.51 | 1.44 | 1.30 | 1.15 | 0.95 | 0.93 | 0.82 | 0.71 |
| WOP | 1.68 | 1.71 | 1.65 | 1.55 | 1.38 | 1.19 | 1.01 | 1.01 | 0.88 | 0.76 |
| PPP | 2.02 | 2.04 | 2.05 | 1.91 | 1.78 | 1.54 | 1.36 | 1.35 | 1.18 | 1.01 |
| PCN | 2.91 | 2.93 | 2.58 | 2.28 | 1.96 | 1.78 | 1.34 | 1.57 | 1.27 | 1.00 |
| CRP | 3.04 | 3.03 | 2.78 | 2.59 | 2.35 | 2.06 | 1.73 | 1.74 | 1.51 | 1.29 |
| NMP | 5.42 | 5.75 | 6.28 | 5.61 | 4.98 | 4.21 | 3.76 | 3.62 | 3.18 | 2.74 |
| MMP | 4.31 | 4.16 | 3.84 | 3.63 | 3.07 | 2.60 | 2.10 | 2.17 | 1.86 | 1.57 |
| TRE | 1.65 | 1.60 | 1.51 | 1.46 | 1.32 | 1.19 | 1.05 | 1.09 | 0.93 | 0.81 |
| EOP | 1.94 | 1.84 | 1.78 | 1.72 | 1.51 | 1.31 | 1.13 | 1.15 | 0.99 | 0.84 |
| OMP | 2.17 | 2.15 | 2.04 | 1.97 | 1.72 | 1.49 | 1.28 | 1.31 | 1.13 | 0.95 |
| EGW | 19.76 | 20.40 | 16.54 | 14.90 | 15.66 | 13.20 | 10.51 | 10.58 | 9.34 | 8.08 |
| SER | 1.53 | 1.58 | 1.57 | 1.47 | 1.30 | 1.12 | 0.95 | 1.58 | 1.39 | 1.29 |

Note: However, the WIOD provides emission data and IO tables only up to 2009. The data of 2010-2011 are extrapolated according to the change ratio of emissions intensity of every major trade partner [14]. Taking the TEI data of trade partner *j* as an example, the TEI for the year 2009 is transformed to that of 2010 and 2011 through the formulae: $F_{j}^{2010}=F_{j}^{2009}\times\frac{\frac{E_{j}^{2010}}{{GDP}_{j}^{2010}}}{\frac{E_{j}^{2009}}{{GDP}_{j}^{2009}}}$,$F_{j}^{2011}=F_{j}^{2010}\times\frac{\frac{E_{j}^{2011}}{{GDP}_{j}^{2011}}}{\frac{E_{j}^{2010}}{{GDP}_{j}^{2010}}}$. Similar data transformations are performed for Table S3-S11.

**Table B. China’s Total CH_4_ Emissions Intensity (2002-2011) (F_ex_ of CH_4_, Ton per US$ Million)**

|  | 2002 | 2003 | 2004 | 2005 | 2006 | 2007 | 2008 | 2009 | 2010 | 2011 |
| --- | --- | --- | --- | --- | --- | --- | --- | --- | --- | --- |
| AGR | 77.24 | 72.41 | 63.10 | 61.16 | 57.30 | 47.39 | 38.11 | 38.01 | 34.67 | 29.81 |
| MIN | 158.32 | 140.89 | 123.43 | 113.30 | 102.93 | 91.92 | 83.19 | 75.08 | 66.85 | 57.65 |
| FOM | 34.01 | 33.28 | 31.24 | 30.62 | 28.69 | 24.08 | 19.25 | 19.47 | 17.32 | 14.85 |
| TLF | 18.12 | 18.35 | 18.21 | 18.22 | 17.05 | 14.58 | 11.86 | 11.96 | 10.52 | 9.02 |
| WOP | 20.74 | 20.73 | 19.96 | 19.09 | 17.29 | 14.17 | 12.11 | 12.54 | 10.84 | 9.15 |
| PPP | 13.09 | 13.54 | 13.24 | 13.12 | 12.48 | 10.63 | 9.06 | 9.30 | 8.05 | 6.83 |
| PCN | 72.05 | 69.77 | 56.48 | 49.14 | 40.21 | 35.16 | 26.53 | 32.16 | 25.91 | 19.96 |
| CRP | 21.39 | 22.10 | 21.11 | 20.18 | 18.47 | 15.83 | 13.36 | 14.02 | 12.14 | 10.25 |
| NMP | 23.60 | 26.57 | 26.38 | 25.59 | 24.06 | 19.99 | 17.32 | 17.20 | 15.37 | 13.31 |
| MMP | 25.90 | 28.09 | 26.12 | 24.70 | 22.98 | 18.08 | 15.19 | 15.75 | 13.42 | 11.27 |
| TRE | 10.53 | 10.56 | 9.82 | 9.67 | 9.14 | 7.84 | 7.03 | 7.51 | 6.36 | 5.47 |
| EOP | 12.00 | 11.94 | 11.27 | 11.05 | 10.20 | 8.46 | 7.43 | 7.76 | 6.62 | 5.53 |
| OMP | 13.93 | 14.21 | 13.14 | 12.81 | 11.84 | 9.73 | 8.56 | 8.88 | 7.63 | 6.34 |
| EGW | 31.65 | 31.15 | 27.80 | 26.34 | 25.07 | 20.95 | 17.92 | 16.62 | 15.70 | 13.51 |
| SER | 17.97 | 17.21 | 15.75 | 14.45 | 12.60 | 10.16 | 8.44 | 8.51 | 7.46 | 6.36 |

**Table C. China’s Total N_2_O Emissions Intensity (2002-2011) (F_ex_ of N_2_O, Ton per US$ Million)**

|  | 2002 | 2003 | 2004 | 2005 | 2006 | 2007 | 2008 | 2009 | 2010 | 2011 |
| --- | --- | --- | --- | --- | --- | --- | --- | --- | --- | --- |
| AGR | 5.47 | 5.10 | 4.45 | 4.25 | 3.97 | 3.24 | 2.57 | 2.56 | 2.31 | 2.06 |
| MIN | 0.20 | 0.18 | 0.16 | 0.15 | 0.14 | 0.12 | 0.10 | 0.11 | 0.09 | 0.08 |
| FOM | 2.17 | 2.11 | 2.00 | 1.94 | 1.81 | 1.50 | 1.18 | 1.19 | 1.05 | 0.93 |
| TLF | 0.96 | 0.95 | 0.95 | 0.93 | 0.88 | 0.73 | 0.58 | 0.58 | 0.51 | 0.45 |
| WOP | 1.02 | 1.02 | 1.01 | 0.95 | 0.86 | 0.69 | 0.57 | 0.60 | 0.51 | 0.44 |
| PPP | 0.49 | 0.50 | 0.49 | 0.48 | 0.46 | 0.39 | 0.32 | 0.33 | 0.28 | 0.24 |
| PCN | 0.24 | 0.20 | 0.17 | 0.15 | 0.13 | 0.11 | 0.09 | 0.11 | 0.08 | 0.07 |
| CRP | 1.05 | 0.91 | 0.78 | 0.68 | 0.59 | 0.47 | 0.37 | 0.39 | 0.33 | 0.29 |
| NMP | 0.31 | 0.29 | 0.26 | 0.24 | 0.22 | 0.19 | 0.16 | 0.17 | 0.14 | 0.12 |
| MMP | 0.24 | 0.22 | 0.18 | 0.17 | 0.16 | 0.14 | 0.11 | 0.12 | 0.10 | 0.09 |
| TRE | 0.23 | 0.20 | 0.17 | 0.16 | 0.15 | 0.13 | 0.11 | 0.12 | 0.10 | 0.09 |
| EOP | 0.25 | 0.22 | 0.19 | 0.19 | 0.17 | 0.14 | 0.12 | 0.13 | 0.11 | 0.09 |
| OMP | 0.30 | 0.26 | 0.21 | 0.20 | 0.19 | 0.16 | 0.14 | 0.14 | 0.12 | 0.10 |
| EGW | 0.49 | 0.49 | 0.41 | 0.39 | 0.42 | 0.37 | 0.32 | 0.33 | 0.28 | 0.25 |
| SER | 0.43 | 0.38 | 0.33 | 0.29 | 0.25 | 0.20 | 0.16 | 0.16 | 0.14 | 0.12 |

**Table D. China’s Total NO_x_ Emissions Intensity (2002-2011) (F_ex_ of NO_x_, Ton per US$ Million)**

|  | 2002 | 2003 | 2004 | 2005 | 2006 | 2007 | 2008 | 2009 | 2010 | 2011 |
| --- | --- | --- | --- | --- | --- | --- | --- | --- | --- | --- |
| AGR | 5.93 | 5.60 | 5.21 | 5.01 | 4.54 | 4.09 | 3.49 | 3.37 | 3.12 | 3.05 |
| MIN | 8.91 | 8.12 | 7.36 | 6.96 | 6.31 | 5.53 | 4.80 | 4.52 | 4.05 | 3.90 |
| FOM | 5.36 | 5.08 | 4.53 | 4.36 | 3.78 | 3.35 | 2.90 | 2.82 | 2.58 | 2.52 |
| TLF | 4.97 | 4.94 | 4.70 | 4.61 | 3.95 | 3.53 | 3.05 | 2.94 | 2.67 | 2.61 |
| WOP | 5.42 | 5.37 | 5.03 | 4.87 | 4.10 | 3.55 | 3.14 | 3.12 | 2.79 | 2.69 |
| PPP | 6.71 | 6.47 | 5.98 | 5.69 | 4.93 | 4.21 | 3.84 | 3.76 | 3.37 | 3.27 |
| PCN | 7.75 | 7.51 | 5.96 | 5.43 | 4.16 | 3.81 | 2.89 | 3.48 | 2.84 | 2.49 |
| CRP | 8.48 | 8.26 | 6.99 | 6.70 | 5.74 | 5.16 | 4.44 | 4.43 | 3.94 | 3.79 |
| NMP | 17.34 | 17.01 | 16.49 | 15.80 | 14.55 | 13.39 | 12.31 | 12.02 | 10.89 | 10.59 |
| MMP | 10.60 | 10.04 | 8.31 | 8.03 | 6.67 | 5.66 | 4.74 | 4.88 | 4.28 | 4.07 |
| TRE | 4.72 | 4.38 | 3.86 | 3.86 | 3.33 | 3.05 | 2.79 | 2.87 | 2.51 | 2.46 |
| EOP | 5.55 | 5.06 | 4.61 | 4.54 | 3.82 | 3.33 | 3.00 | 3.02 | 2.67 | 2.55 |
| OMP | 6.04 | 5.78 | 5.19 | 5.15 | 4.28 | 3.74 | 3.32 | 3.35 | 2.97 | 2.81 |
| EGW | 40.84 | 42.63 | 44.09 | 43.65 | 42.33 | 36.30 | 27.95 | 27.32 | 24.82 | 24.19 |
| SER | 5.78 | 5.72 | 4.82 | 4.55 | 3.84 | 3.41 | 3.15 | 3.10 | 2.80 | 2.72 |

**Table E. China’s Total SO_x_ Emissions Intensity (2002-2011) (F_ex_ of SO_x_, Ton per US$ Million)**

|  | 2002 | 2003 | 2004 | 2005 | 2006 | 2007 | 2008 | 2009 | 2010 | 2011 |
| --- | --- | --- | --- | --- | --- | --- | --- | --- | --- | --- |
| AGR | 7.05 | 6.38 | 5.47 | 4.90 | 4.11 | 3.60 | 2.99 | 2.86 | 2.38 | 1.95 |
| MIN | 20.30 | 19.23 | 17.66 | 15.12 | 12.55 | 10.76 | 7.77 | 6.60 | 5.33 | 4.31 |
| FOM | 9.41 | 8.23 | 6.76 | 5.99 | 4.97 | 4.17 | 3.21 | 2.93 | 2.41 | 1.98 |
| TLF | 10.27 | 9.64 | 8.80 | 8.17 | 6.76 | 5.65 | 4.23 | 3.73 | 3.06 | 2.52 |
| WOP | 12.06 | 11.12 | 9.79 | 8.90 | 7.28 | 5.92 | 4.52 | 4.07 | 3.29 | 2.67 |
| PPP | 16.26 | 15.42 | 13.21 | 11.93 | 9.88 | 7.76 | 5.91 | 4.95 | 4.00 | 3.27 |
| PCN | 25.33 | 21.43 | 17.17 | 14.21 | 11.21 | 9.62 | 6.51 | 6.40 | 4.81 | 3.64 |
| CRP | 19.84 | 18.54 | 15.87 | 14.07 | 11.61 | 10.22 | 7.97 | 7.28 | 5.85 | 4.75 |
| NMP | 52.19 | 50.30 | 37.82 | 33.08 | 29.56 | 17.09 | 12.88 | 11.43 | 9.29 | 7.58 |
| MMP | 37.12 | 32.43 | 26.94 | 23.12 | 20.17 | 13.04 | 9.88 | 8.87 | 7.05 | 5.67 |
| TRE | 13.08 | 11.57 | 9.78 | 8.86 | 7.69 | 5.87 | 4.68 | 4.30 | 3.40 | 2.80 |
| EOP | 14.95 | 12.98 | 11.54 | 10.27 | 8.65 | 6.52 | 5.02 | 4.51 | 3.59 | 2.88 |
| OMP | 16.64 | 15.18 | 13.23 | 11.94 | 9.96 | 7.47 | 5.78 | 5.15 | 4.12 | 3.28 |
| EGW | 142.92 | 136.43 | 110.89 | 95.12 | 85.36 | 74.20 | 51.35 | 43.62 | 35.68 | 29.27 |
| SER | 11.40 | 10.67 | 9.09 | 8.05 | 6.72 | 4.99 | 3.80 | 3.41 | 2.75 | 2.25 |

**Table F. Total CO_2_ Emissions Intensity of China’s major trade partners (2002-2011) (F_tp_ of CO_2_, Kt per US$ Million)**

|  | 2002 | 2003 | 2004 | 2005 | 2006 | 2007 | 2008 | 2009 | 2010 | 2011 |
| --- | --- | --- | --- | --- | --- | --- | --- | --- | --- | --- |
| AGR | 0.69 | 0.65 | 0.60 | 0.59 | 0.53 | 0.45 | 0.39 | 0.40 | 0.36 | 0.32 |
| MIN | 1.94 | 1.89 | 1.78 | 1.64 | 1.45 | 1.37 | 1.22 | 1.20 | 1.11 | 0.99 |
| FOM | 0.68 | 0.65 | 0.62 | 0.61 | 0.55 | 0.50 | 0.44 | 0.44 | 0.40 | 0.35 |
| TLF | 0.91 | 0.94 | 0.92 | 0.92 | 0.85 | 0.78 | 0.62 | 0.64 | 0.57 | 0.50 |
| WOP | 0.83 | 0.80 | 0.77 | 0.74 | 0.68 | 0.61 | 0.55 | 0.57 | 0.51 | 0.46 |
| PPP | 1.00 | 1.02 | 0.99 | 0.97 | 0.91 | 0.82 | 0.75 | 0.76 | 0.68 | 0.61 |
| PCN | 1.59 | 1.45 | 1.25 | 1.11 | 0.96 | 0.89 | 0.69 | 0.86 | 0.75 | 0.61 |
| CRP | 1.46 | 1.38 | 1.26 | 1.22 | 1.12 | 1.02 | 0.90 | 0.93 | 0.82 | 0.73 |
| NMP | 3.96 | 3.92 | 3.90 | 3.68 | 3.37 | 3.04 | 2.73 | 2.72 | 2.47 | 2.17 |
| MMP | 2.36 | 2.31 | 2.00 | 1.93 | 1.85 | 1.57 | 1.28 | 1.37 | 1.23 | 1.08 |
| TRE | 0.78 | 0.73 | 0.67 | 0.66 | 0.62 | 0.56 | 0.52 | 0.54 | 0.47 | 0.43 |
| EOP | 0.79 | 0.75 | 0.72 | 0.71 | 0.66 | 0.59 | 0.54 | 0.56 | 0.50 | 0.44 |
| OMP | 0.92 | 0.89 | 0.83 | 0.82 | 0.75 | 0.67 | 0.61 | 0.64 | 0.57 | 0.50 |
| EGW | 10.21 | 9.90 | 8.80 | 8.48 | 8.39 | 7.56 | 6.56 | 6.61 | 6.03 | 5.38 |
| SER | 0.70 | 0.69 | 0.68 | 0.66 | 0.60 | 0.54 | 0.48 | 0.49 | 0.44 | 0.39 |

**Table G. Total CH_4_ Emissions Intensity of China’s major trade partners (2002-2011) (F_tp_ of CH_4_, Ton per US$ Million)**

|  | 2002 | 2003 | 2004 | 2005 | 2006 | 2007 | 2008 | 2009 | 2010 | 2011 |
| --- | --- | --- | --- | --- | --- | --- | --- | --- | --- | --- |
| AGR | 42.71 | 39.42 | 37.09 | 38.12 | 35.85 | 30.13 | 26.45 | 27.17 | 24.87 | 22.66 |
| MIN | 53.59 | 48.80 | 43.85 | 41.22 | 38.86 | 35.99 | 34.87 | 32.55 | 29.49 | 26.85 |
| FOM | 15.71 | 15.02 | 14.77 | 15.59 | 14.22 | 12.08 | 10.64 | 10.89 | 9.92 | 9.03 |
| TLF | 6.54 | 6.55 | 6.48 | 6.91 | 6.42 | 5.68 | 4.99 | 4.99 | 4.57 | 4.18 |
| WOP | 9.72 | 9.03 | 8.65 | 8.73 | 7.92 | 6.61 | 6.08 | 6.68 | 5.82 | 5.30 |
| PPP | 5.04 | 4.93 | 4.78 | 4.94 | 4.69 | 4.15 | 3.81 | 3.91 | 3.47 | 3.17 |
| PCN | 20.33 | 20.01 | 16.93 | 15.68 | 13.49 | 12.36 | 10.36 | 12.22 | 10.31 | 8.65 |
| CRP | 7.69 | 7.81 | 7.35 | 7.43 | 6.95 | 6.27 | 5.68 | 5.95 | 5.29 | 4.77 |
| NMP | 8.84 | 9.39 | 9.21 | 9.27 | 8.86 | 7.76 | 7.19 | 7.21 | 6.53 | 6.03 |
| MMP | 8.72 | 9.40 | 8.76 | 8.71 | 8.34 | 7.10 | 6.31 | 6.62 | 5.79 | 5.24 |
| TRE | 4.26 | 4.02 | 3.65 | 3.74 | 3.53 | 3.11 | 2.89 | 3.04 | 2.64 | 2.43 |
| EOP | 3.83 | 3.78 | 3.64 | 3.78 | 3.55 | 3.07 | 2.88 | 3.03 | 2.66 | 2.39 |
| OMP | 5.43 | 5.31 | 4.92 | 4.95 | 4.66 | 3.94 | 3.58 | 3.83 | 3.37 | 3.02 |
| EGW | 17.03 | 14.87 | 12.80 | 12.33 | 11.27 | 9.87 | 8.89 | 8.68 | 8.05 | 7.43 |
| SER | 7.79 | 7.30 | 6.85 | 6.67 | 5.77 | 4.86 | 4.28 | 4.34 | 3.85 | 3.51 |

**Table H. Total N_2_O Emissions Intensity of China’s major trade partners (2002-2011) (F_tp_ of N_2_O, Ton per US$ Million)**

|  | 2002 | 2003 | 2004 | 2005 | 2006 | 2007 | 2008 | 2009 | 2010 | 2011 |
| --- | --- | --- | --- | --- | --- | --- | --- | --- | --- | --- |
| AGR | 3.20 | 2.64 | 2.72 | 2.92 | 2.64 | 2.08 | 1.78 | 1.83 | 1.64 | 1.53 |
| MIN | 0.06 | 0.06 | 0.05 | 0.05 | 0.05 | 0.05 | 0.04 | 0.04 | 0.04 | 0.03 |
| FOM | 1.05 | 0.95 | 0.98 | 1.05 | 0.95 | 0.76 | 0.66 | 0.67 | 0.60 | 0.56 |
| TLF | 0.31 | 0.31 | 0.31 | 0.34 | 0.32 | 0.27 | 0.23 | 0.23 | 0.21 | 0.19 |
| WOP | 0.54 | 0.48 | 0.48 | 0.49 | 0.43 | 0.34 | 0.31 | 0.34 | 0.29 | 0.27 |
| PPP | 0.19 | 0.18 | 0.18 | 0.19 | 0.18 | 0.15 | 0.14 | 0.14 | 0.12 | 0.12 |
| PCN | 0.07 | 0.16 | 0.05 | 0.05 | 0.04 | 0.04 | 0.04 | 0.04 | 0.03 | 0.03 |
| CRP | 0.41 | 0.35 | 0.34 | 0.32 | 0.28 | 0.23 | 0.20 | 0.21 | 0.19 | 0.17 |
| NMP | 0.12 | 0.11 | 0.10 | 0.10 | 0.10 | 0.08 | 0.08 | 0.08 | 0.07 | 0.07 |
| MMP | 0.09 | 0.08 | 0.07 | 0.07 | 0.07 | 0.06 | 0.05 | 0.06 | 0.05 | 0.04 |
| TRE | 0.09 | 0.08 | 0.07 | 0.07 | 0.07 | 0.06 | 0.05 | 0.05 | 0.05 | 0.04 |
| EOP | 0.08 | 0.07 | 0.07 | 0.07 | 0.07 | 0.06 | 0.05 | 0.05 | 0.05 | 0.04 |
| OMP | 0.15 | 0.15 | 0.12 | 0.12 | 0.11 | 0.09 | 0.07 | 0.08 | 0.07 | 0.06 |
| EGW | 0.21 | 0.20 | 0.19 | 0.19 | 0.19 | 0.17 | 0.16 | 0.16 | 0.15 | 0.13 |
| SER | 0.16 | 0.15 | 0.14 | 0.14 | 0.12 | 0.09 | 0.08 | 0.08 | 0.07 | 0.07 |

**Table I. Total NO_x_ Emissions Intensity of China’s major trade partners (2002-2011) (F_tp_ of NO_x_, Ton per US$ Million)**

|  | 2002 | 2003 | 2004 | 2005 | 2006 | 2007 | 2008 | 2009 | 2010 | 2011 |
| --- | --- | --- | --- | --- | --- | --- | --- | --- | --- | --- |
| AGR | 4.80 | 4.16 | 4.16 | 4.15 | 3.98 | 3.51 | 3.05 | 3.11 | 2.82 | 2.68 |
| MIN | 5.49 | 5.08 | 4.55 | 4.22 | 3.77 | 3.24 | 3.02 | 3.03 | 2.71 | 2.49 |
| FOM | 3.22 | 2.88 | 3.31 | 3.34 | 3.10 | 2.63 | 2.16 | 2.28 | 1.99 | 1.81 |
| TLF | 3.39 | 3.29 | 2.98 | 3.12 | 2.79 | 2.23 | 1.93 | 1.96 | 1.77 | 1.65 |
| WOP | 3.62 | 3.24 | 3.13 | 3.03 | 2.80 | 2.39 | 2.11 | 2.34 | 1.99 | 1.84 |
| PPP | 3.74 | 3.53 | 3.21 | 3.23 | 2.91 | 2.39 | 2.24 | 2.28 | 2.04 | 1.94 |
| PCN | 3.55 | 3.25 | 2.50 | 2.36 | 1.94 | 1.73 | 1.42 | 1.76 | 1.51 | 1.30 |
| CRP | 4.09 | 3.76 | 3.10 | 3.05 | 2.75 | 2.45 | 2.23 | 2.28 | 2.02 | 1.93 |
| NMP | 11.72 | 11.69 | 10.13 | 9.76 | 9.14 | 8.02 | 7.96 | 8.04 | 7.33 | 6.90 |
| MMP | 5.43 | 5.12 | 3.89 | 3.84 | 3.57 | 2.83 | 2.52 | 2.71 | 2.40 | 2.24 |
| TRE | 2.47 | 2.18 | 1.80 | 1.83 | 1.66 | 1.44 | 1.39 | 1.44 | 1.27 | 1.24 |
| EOP | 2.41 | 2.18 | 1.93 | 1.95 | 1.76 | 1.50 | 1.45 | 1.51 | 1.34 | 1.27 |
| OMP | 2.84 | 2.62 | 2.25 | 2.26 | 2.01 | 1.71 | 1.61 | 1.69 | 1.50 | 1.43 |
| EGW | 25.08 | 23.58 | 21.24 | 21.79 | 20.57 | 17.96 | 15.55 | 15.34 | 14.05 | 13.40 |
| SER | 3.17 | 2.93 | 2.42 | 2.35 | 2.06 | 1.83 | 1.78 | 1.82 | 1.63 | 1.54 |

**Table J. Total SO_x_ Emissions Intensity of China’s major trade partners (2002-2011) (F_tp_ of SO_x_, Ton per US$ Million)**

|  | 2002 | 2003 | 2004 | 2005 | 2006 | 2007 | 2008 | 2009 | 2010 | 2011 |
| --- | --- | --- | --- | --- | --- | --- | --- | --- | --- | --- |
| AGR | 2.88 | 2.60 | 3.47 | 3.59 | 3.39 | 3.18 | 2.49 | 2.57 | 2.12 | 1.74 |
| MIN | 11.19 | 11.21 | 13.93 | 12.79 | 12.08 | 6.57 | 5.11 | 3.62 | 3.10 | 2.60 |
| FOM | 3.58 | 3.18 | 3.08 | 2.99 | 2.68 | 2.41 | 1.96 | 1.97 | 1.61 | 1.34 |
| TLF | 4.90 | 4.76 | 4.07 | 3.89 | 3.40 | 2.76 | 2.30 | 2.16 | 1.84 | 1.56 |
| WOP | 4.57 | 4.20 | 3.89 | 3.72 | 3.32 | 2.75 | 2.30 | 2.31 | 1.87 | 1.58 |
| PPP | 6.21 | 6.10 | 5.04 | 4.90 | 4.21 | 3.30 | 2.86 | 2.55 | 2.15 | 1.84 |
| PCN | 9.52 | 7.95 | 6.34 | 5.42 | 4.66 | 4.09 | 3.19 | 3.42 | 2.79 | 2.20 |
| CRP | 6.66 | 6.46 | 5.47 | 5.21 | 4.58 | 4.22 | 3.69 | 3.45 | 2.87 | 2.43 |
| NMP | 17.45 | 17.18 | 13.19 | 12.52 | 11.72 | 8.27 | 7.54 | 7.11 | 6.13 | 5.24 |
| MMP | 12.24 | 11.06 | 8.76 | 8.15 | 7.37 | 5.20 | 4.55 | 4.30 | 3.60 | 3.04 |
| TRE | 4.31 | 3.89 | 3.22 | 3.10 | 2.81 | 2.27 | 2.03 | 1.88 | 1.55 | 1.35 |
| EOP | 4.51 | 4.05 | 3.62 | 3.43 | 3.05 | 2.43 | 2.12 | 1.95 | 1.62 | 1.37 |
| OMP | 5.05 | 4.75 | 4.16 | 3.99 | 3.49 | 2.77 | 2.42 | 2.21 | 1.85 | 1.56 |
| EGW | 74.42 | 67.86 | 49.39 | 44.71 | 40.78 | 38.62 | 31.55 | 29.10 | 25.26 | 21.54 |
| SER | 3.68 | 3.47 | 2.96 | 2.79 | 2.42 | 1.92 | 1.67 | 1.52 | 1.28 | 1.10 |

**REFERENCE**

1. Lin J, Pan D, Davis SJ, Zhang Q, He K, et al. (2014) China’s International Trade and Air Pollution in the United States. Proc. Natl. Acad. Sci. 111: 1736–1741.
