# Supplementary material for: GHGs and air pollutants embodied in China’s international trade: Temporal and spatial index decomposition analysis
Source: PLoS One. 2017 Apr 25;12(4):e0176089. doi: 10.1371/journal.pone.0176089 (PMC5404823; doi:10.1371/journal.pone.0176089)
Supplement: S4 Text — (DOCX) [file pone.0176089.s004.docx]

## S4 Text. Uncertainty analysis of decomposition results

For the uncertainty analysis of the temporal IDA of EEEs, the export scale data was selected to perturb and re-compute the estimates for total scale, composition and technique effects. Selection of export was due to its dominant contribution to the changes in EEEs. Data of 2004, 2007 and 2010 were selected for the sampling. When expanding or decreasing export by 5%, which is a significant perturbation in the circumstance of China’s annual export value, the change rates of the total scale, composition and technique effects for the pollutants, GHGs, NO_x_, SO_x_, did not surpass 1% in general. (see **Table A**)

For the uncertainty analysis of the spatial IDA for BEETs, the emission intensity data was selected to perturb also due to its dominating role in driving BEETs. When increasing or decreasing emission intensity by 5%, which is also a significant perturbation in the circumstance of China’s emission intensity, for the 3 sampling years, the changes in intensity effect, specialization effect and trade balance effect varied between 1.40-7.42%. (see **Table B**)

So the decomposition results of the paper are not greatly affected by such major perturbations of the data and thus the robustness of the decompositions results is confirmed.

**Table A. Uncertainty analysis of the temporal IDA for EEEs**

| Sampling years | 2004 | | 2007 | | 2010 | |
| --- | --- | --- | --- | --- | --- | --- |
| Percentage change | +5% | -5% | +5% | -5% | +5% | -5% |
| GHGs (%) | | | | | | |
| ${\Delta EEE}_{scl}$ | 0.11 | -0.11 | 0.56 | -0.57 | 0.50 | -0.51 |
| ${\Delta EEE}_{comp}$ | 0.02 | -0.02 | 0.05 | -0.05 | 0.06 | -0.06 |
| ${\Delta EEE}_{tch}$ | 0.21 | -0.21 | 1.04 | -1.06 | 0.94 | -0.95 |
| NO_x_ (%) | | | | | | |
| ${\Delta EEE}_{scl}$ | 0.14 | -0.15 | 0.43 | -0.44 | 0.25 | -0.26 |
| ${\Delta EEE}_{comp}$ | 0.02 | -0.02 | 0.03 | -0.03 | 0.04 | -0.04 |
| ${\Delta EEE}_{tch}$ | 0.33 | -0.34 | 0.96 | -0.97 | 0.58 | -0.59 |
| SO_x_ (%) | | | | | | |
| ${\Delta EEE}_{scl}$ | 0.32 | -0.33 | 0.91 | -0.92 | 0.49 | -0.50 |
| ${\Delta EEE}_{comp}$ | 0.66 | -0.67 | 0.46 | -0.46 | 0.31 | -0.32 |
| ${\Delta EEE}_{tch}$ | 0.42 | -0.36 | 0.98 | -1.00 | 0.54 | -0.55 |

**Table B. Uncertainty analysis of the spatial IDA for BEEEs**

| Sampling years  Percentage change | 2004 | | 2007 | | 2010 | |
| --- | --- | --- | --- | --- | --- | --- |
|  | +5% | -5% | +5% | -5% | +5% | -5% |
| GHGs (%) | | | | | | |
| ΔEI | 6.28 | -6.29 | 6.39 | -6.40 | 6.37 | -6.38 |
| ΔSP | 1.48 | -1.50 | 1.49 | -1.51 | 1.44 | -1.46 |
| ΔTB | 1.48 | -1.50 | 1.49 | -1.51 | 1.44 | -1.46 |
| SO_x_ (%) | | | | | | |
| ΔEI | 6.05 | -6.07 | 5.98 | -5.99 | 6.24 | -6.26 |
| ΔSP | 1.46 | -1.48 | 1.48 | -1.50 | 1.42 | -1.44 |
| ΔTB | 1.46 | -1.48 | 1.48 | -1.50 | 1.42 | -1.44 |
| NO_x_ (%) | | | | | | |
| ΔEI | 7.41 | -7.42 | 6.79 | -6.80 | 6.55 | -6.56 |
| ΔSP | 1.43 | -1.45 | 1.45 | -1.47 | 1.40 | -1.42 |
| ΔTB | 1.43 | -1.45 | 1.45 | -1.47 | 1.40 | -1.42 |
