# Supplementary material for: GHGs and air pollutants embodied in China’s international trade: Temporal and spatial index decomposition analysis
Source: PLoS One. 2017 Apr 25;12(4):e0176089. doi: 10.1371/journal.pone.0176089 (PMC5404823; doi:10.1371/journal.pone.0176089)
Supplement: S1 Table — (DOCX) [file pone.0176089.s005.docx]

**S1 Table. Sector classification for the current study**

| Aggregated sectors used in this study | Sectors in the EIO model of WIOD |
| --- | --- |
| 1 Agriculture (AGR) | Agriculture, Hunting, Forestry and Fishing |
| 2 Mining (MIN) | Mining and Quarrying |
| 3 Food manufacturing (FOM) | Food, Beverages and Tobacco |
| 4 Textiles, leather and footwear manufacturing (TLF) | Textiles and Textile Products; Leather, Leather and Footwear |
| 5 Wood and Products of Wood and Cork (WOP) | Wood and Products of Wood and Cork |
| 6 Paper and publishing products (PPP) | Pulp, Paper, Paper , Printing and Publishing |
| 7 Petroleum processing, coking and nuclear fuel (PCN) | Coke, Refined Petroleum and Nuclear Fuel |
| 8 Chemicals and Chemical Products; Rubber and Plastics (CRP) | Chemicals and Chemical Products; Rubber and Plastics |
| 9 Non-metal mineral products (NMP) | Other Non-Metallic Mineral |
| 10 Metals and Metal productions (MMP) | Basic Metals and Fabricated Metal |
| 11 Transport equipment (TRE) | Transport equipment |
| 12 Electrical and Optical Equipment (EOP) | Electrical and Optical Equipment |
| 13 Other manufacturing products (OMP) | Machinery, Nec; Manufacturing, Nec; Recycling |
| 14 Electricity, gas and water production and supply (EGW) | Electricity, Gas and Water Supply |
| 15 Services (SER) | Construction; Sale, Maintenance and Repair of Motor Vehicles and Motorcycles; Retail Sale of Fuel; Wholesale Trade and Commission Trade, Except of Motor Vehicles and Motorcycles; Retail Trade, Except of Motor Vehicles and Motorcycles; Repair of Household Goods; Hotels and Restaurants; Inland Transport; Water Transport; Air Transport; Other Supporting and Auxiliary Transport Activities; Activities of Travel Agencies; Post and Telecommunications; Financial Intermediation; Real Estate Activities; Renting of M&Eq and Other Business Activities; Public Admin and Defence; Compulsory Social Security; Education; Health and Social Work; Other Community, Social and Personal Services; Private Households with Employed Persons |
