# Supplementary material for: GHGs and air pollutants embodied in China’s international trade: Temporal and spatial index decomposition analysis
Source: PLoS One. 2017 Apr 25;12(4):e0176089. doi: 10.1371/journal.pone.0176089 (PMC5404823; doi:10.1371/journal.pone.0176089)
Supplement: S2 Table — Note: * Average value of the calculation years. (DOCX) [file pone.0176089.s006.docx]

**S2 Table. A Comparison of EEEs and BEETs of China in this study with previous studies**

| Environmental performance indicator | Pollutants | Proportion in the total annual emission of China (%) | Calculation years | Literature |
| --- | --- | --- | --- | --- |
| EEE | SO_2_ | 15.17  22.08 | 2002  2007 | Liu and Wang [21] |
|  |  | 32.01* | 2002-2011 | This study |
| EEE | GHGs | 48* | 2002-2008 | Xu et al. [32] |
|  |  | 21 | 2002 | Weber et al. [20] |
|  |  | 33 | 2005 |  |
|  |  | 22.1 | 2007 | Liu et al. [69] |
|  |  | 26.54 | 2007 | Yan and Yang [23] |
|  |  | 31 | 2007 | Weitzel and Ma [44] |
|  |  | 28.96* | 2002-2011 | This study |
| BEET | GHGs | 42  24 | 2007  2011 | Ren et al. [24] |
|  |  | 22.6 | 2004 | Davis and Caldeira [12] |
|  |  | 22 | 2007 | Qi et al. [9] |
|  |  | 27.3 | 2007 | Aichele and Felbermayr [70] |
|  |  | 19.3 | 2007 | Liu et al. [69] |
|  |  | 17.49 | 2007 | Yan and Yang [23] |
|  |  | 19.66* | 2002-2011 | This study |

Note: * Average value of the calculation years.
