# Supplementary material for: GHGs and air pollutants embodied in China’s international trade: Temporal and spatial index decomposition analysis
Source: PLoS One. 2017 Apr 25;12(4):e0176089. doi: 10.1371/journal.pone.0176089 (PMC5404823; doi:10.1371/journal.pone.0176089)
Supplement: S3 Table — (DOCX) [file pone.0176089.s007.docx]

**S3 Table. Decomposition of pollutants embodied in China’s net exports (BEET) during 2002–2011**

|  | 2002 | 2003 | 2004 | 2005 | 2006 | 2007 | 2008 | 2009 | 2010 | 2011 | Total |
| --- | --- | --- | --- | --- | --- | --- | --- | --- | --- | --- | --- |
| GHGs (Mt, CO_2_-eq) | | | | | | | | | | | |
| BEET | 719.6 | 923.5 | 1200.1 | 1497.9 | 1717.6 | 1843.8 | 1801.6 | 1431.5 | 1559.8 | 1530.5 | 14225.9 |
| ΔEI | 885.1 | 1152.0 | 1534.7 | 1819.0 | 2033.9 | 2179.3 | 2325.6 | 1950.3 | 2260.0 | 2310.5 | 18450.4 |
| $\Delta SP$ | -222.6 | -275.7 | -380.6 | -494.5 | -632.4 | -754.8 | -1021.3 | -741.2 | -1056.0 | -1132.4 | -6711.6 |
| $\Delta T$B | 57.1 | 47.2 | 46.0 | 173.4 | 316.1 | 419.4 | 497.3 | 222.5 | 355.8 | 352.4 | 2487.2 |
| SO_x_ (Kt) | | | | | | | | | | | |
| BEET | 4075.4 | 4755.9 | 5511.3 | 6319.0 | 6780.8 | 6741.2 | 5738.8 | 4056.0 | 4066.0 | 3749.9 | 51794.2 |
| ΔEI | 5083.8 | 6451.0 | 8079.8 | 9656.4 | 10826.1 | 9620.5 | 9174.1 | 6138.2 | 6766.0 | 7037.3 | 78833.1 |
| $\Delta SP$ | -1320.0 | -1939.3 | -2796.4 | -4163.0 | -5512.4 | -4526.4 | -5248.2 | -2781.5 | -3741.0 | -4267.2 | -36295.3 |
| $\Delta T$B | 311.6 | 244.2 | 227.9 | 825.5 | 1467.1 | 1647.1 | 1812.9 | 699.3 | 1041.0 | 979.7 | 9256.5 |
| NO_x_ (Kt) | | | | | | | | | | | |
| BEET | 1336.2 | 1685.3 | 2115.4 | 2748.1 | 2973.6 | 3410.3 | 3377.8 | 2600.9 | 2941.2 | 3362.5 | 26551.3 |
| ΔEI | 1484.9 | 1986.3 | 2563.0 | 3302.9 | 3684.8 | 4270.4 | 4833.8 | 4027.4 | 5009.5 | 5865.4 | 37028.3 |
| $\Delta SP$ | -294.8 | -417.3 | -553.1 | -959.5 | -1423.9 | -1798.6 | -2621.3 | -1951.2 | -2917.8 | -3424.1 | -16361.6 |
| $\Delta T$B | 146.2 | 116.2 | 105.5 | 404.7 | 712.8 | 938.5 | 1165.3 | 524.7 | 849.6 | 921.2 | 5884.6 |
