# Supplementary material for: GHGs and air pollutants embodied in China’s international trade: Temporal and spatial index decomposition analysis
Source: PLoS One. 2017 Apr 25;12(4):e0176089. doi: 10.1371/journal.pone.0176089 (PMC5404823; doi:10.1371/journal.pone.0176089)
Supplement: S4 Table — Note: EIc/EItp is the economy-wide emissions intensity of China divided by its trade partners; spc/sptp is the degree of ‘pollution intensive product specialization’ of exports of China divided by that of its trade partners. (DOCX) [file pone.0176089.s008.docx]

**S4 Table. Yearly ratio of EI_c_/EI_tp_, sp_c_/sp_tp_ and X/M during 2002-2011**

|  | 2002 | 2003 | 2004 | 2005 | 2006 | 2007 | 2008 | 2009 | 2010 | 2011 |
| --- | --- | --- | --- | --- | --- | --- | --- | --- | --- | --- |
| GHGs | | | | | | | | | | |
| EI_c_ (Mt/ US$ billion) | 2.40 | 2.36 | 2.41 | 2.34 | 2.29 | 2.19 | 2.22 | 2.13 | 2.10 | 2.05 |
| EI_tp_ (Mt/ US$ billion) | 0.56 | 0.54 | 0.53 | 0.51 | 0.51 | 0.49 | 0.5 | 0.49 | 0.5 | 0.51 |
| EI_c_/EI_tp_ | 4.32 | 4.34 | 4.52 | 4.55 | 4.51 | 4.45 | 4.42 | 4.37 | 4.24 | 4.03 |
| sp_c_ | 1.00 | 1.10 | 1.17 | 1.18 | 1.01 | 0.93 | 0.85 | 0.82 | 0.74 | 0.71 |
| sp_tp_ | 1.45 | 1.57 | 1.7 | 1.79 | 1.6 | 1.55 | 1.67 | 1.44 | 1.45 | 1.39 |
| sp_c_/sp_tp_ | 0.69 | 0.70 | 0.69 | 0.66 | 0.63 | 0.60 | 0.51 | 0.57 | 0.51 | 0.51 |
| X/M | 1.10 | 1.06 | 1.05 | 1.15 | 1.26 | 1.33 | 1.38 | 1.18 | 1.26 | 1.24 |
| SO_x_ | | | | | | | | | | |
| EI_c_ (Kt/ US$ billion) | 10.14 | 9.74 | 9.05 | 9.09 | 8.57 | 7.26 | 6.80 | 5.85 | 5.16 | 4.99 |
| EI_tp_ (Kt/ US$ billion) | 2.14 | 2 | 1.81 | 1.7 | 1.55 | 1.38 | 1.32 | 1.3 | 1.18 | 1.08 |
| EI_c_/EI_tp_ | 4.73 | 4.88 | 4.99 | 5.36 | 5.54 | 5.25 | 5.15 | 4.48 | 4.38 | 4.60 |
| sp_c_ | 1.31 | 1.37 | 1.50 | 1.36 | 1.16 | 1.07 | 0.95 | 0.90 | 0.84 | 0.77 |
| sp_tp_ | 1.96 | 2.21 | 2.63 | 2.83 | 2.83 | 2.33 | 2.44 | 1.76 | 1.91 | 1.93 |
| sp_c_/sp_tp_ | 0.67 | 0.62 | 0.57 | 0.48 | 0.41 | 0.46 | 0.39 | 0.51 | 0.44 | 0.40 |
| X/M | 1.10 | 1.06 | 1.05 | 1.15 | 1.26 | 1.33 | 1.38 | 1.18 | 1.26 | 1.24 |
| NO_x_ | | | | | | | | | | |
| EI_c_ (Kt/ US$ billion) | 6.96 | 6.96 | 6.89 | 6.84 | 6.58 | 6.58 | 6.40 | 5.95 | 5.79 | 5.51 |
| EI_tp_ (Kt/ US$ billion) | 2.55 | 2.41 | 2.22 | 2.05 | 1.9 | 1.81 | 1.76 | 1.63 | 1.56 | 1.46 |
| EI_c_/EI_tp_ | 2.73 | 2.89 | 3.11 | 3.33 | 3.46 | 3.64 | 3.63 | 3.64 | 3.72 | 3.77 |
| sp_c_ | 0.77 | 0.81 | 0.84 | 0.84 | 0.70 | 0.64 | 0.63 | 0.62 | 0.59 | 0.65 |
| sp_tp_ | 0.93 | 1 | 1.06 | 1.18 | 1.11 | 1.1 | 1.3 | 1.17 | 1.28 | 1.44 |
| sp_c_/sp_tp_ | 0.83 | 0.81 | 0.79 | 0.71 | 0.63 | 0.58 | 0.48 | 0.53 | 0.46 | 0.45 |
| X/M | 1.10 | 1.06 | 1.05 | 1.15 | 1.26 | 1.33 | 1.38 | 1.18 | 1.26 | 1.24 |

Note: EI_c_/EI_tp_ is the economy-wide emissions intensity of China divided by its trade partners; sp_c_/sp_tp_ is the degree of ‘pollution intensive product specialization’ of exports of China divided by that of its trade partners.
